# Tolerance and dose-response assessment of subchronic dietary ethoxyquin exposure in Atlantic salmon (*Salmo salar* L.)

**DOI:** 10.1371/journal.pone.0211128

**Published:** 2019-01-25

**Authors:** Annette Bernhard, Josef D. Rasinger, Monica B. Betancor, Maria José Caballero, Marc H. G. Berntssen, Anne-Katrine Lundebye, Robin Ørnsrud

**Affiliations:** 1 Institute of Marine Research, Bergen, Norway; 2 Institute of Aquaculture, Faculty of Natural Sciences, University of Stirling, Stirling, United Kingdom; 3 Department of Morphology, Veterinary School, University of Las Palmas de Gran Canaria, Las Palmas de Gran Canaria, Spain; University of Illinois, UNITED STATES

## Abstract

Ethoxyquin (EQ; 6-Ethoxy-2,2,4-trimethyl-1,2-dihydroquinoline) has been used as an antioxidant in feed components for pets, livestock and aquaculture. However, possible risks of EQ used in aquafeed for fish health have not yet been characterized. The present study investigated the toxicity and dose-response of subchronic dietary EQ exposure at doses ranging from 41 to 9666 mg EQ/kg feed in Atlantic salmon (*Salmo salar* L.). Feed at concentrations higher than 1173 mg EQ/kg were rejected by the fish, resulting in reduced feed intake and growth performance. No mortality was observed in fish exposed to any of the doses. A multi-omic screening of metabolome and proteome in salmon liver indicated an effect of dietary EQ on bioenergetics pathways and hepatic redox homeostasis in fish fed concentrations above 119 mg EQ/kg feed. Increased energy expenditure associated with an upregulation of hepatic fatty acid β-oxidation and induction and carbohydrate catabolic pathways resulted in a dose-dependent depletion of intracytoplasmic lipid vacuoles in liver histological sections, decreasing whole body lipid levels and altered purine/pyrimidine metabolism. Increased GSH and TBARS in the liver indicated a state of oxidative stress, which was associated with activation of the NRF2-mediated oxidative stress response and glutathione-mediated detoxification processes. However, no oxidative DNA damage was observed. As manifestation of altered energy metabolism, the depletion of liver intracytoplasmic lipid vacuoles was considered the critical endpoint for benchmark dose assessment, and a BMDL_10_ of 243 mg EQ/kg feed was derived as a safe upper limit of EQ exposure in Atlantic salmon.

## Introduction

During storage, animal feed may be subject to auto-oxidation induced lipid peroxidation, which can result in feed rancidity and reduced shelf life. Ethoxyquin (6-Ethoxy-2,2,4-trimethyl-1,2-dihydroquinoline; EQ) is one of a number of technological antioxidant feed additives added to animal feeds to prevent this process from happening. Because of its high antioxidant capacity [1], EQ has commonly been used in fishmeal to prevent rancidity, but also to prevent self-ignition under long-distance sea transport and storage. In addition, EQ has routinely been added to fish silage as well as vitamin and pigment premixes in order to preserve nutritive value, freshness, flavor and color of these products. In the European Union (EU), the upper limit (UL) for use of EQ alone or in combination with two other authorized synthetic antioxidants, namely butylated hydroxy toluene (BHT) and butylated hydroxyanisole (BHA), in animal feed was previously established at 150 mg/kg (Council Directive 70/524/EEC, replaced by Council Regulation (EC) No. 1831/2003 [2,3]). Results from an annual feed monitoring program commissioned by the Norwegian Food Safety Authorities showed that during the years 2003–2017 the average EQ concentration in Norwegian fish feeds was 20 ± 25 mg/kg (including both EQ and its main metabolite ethoxyquin dimer (EQDM); *n* = 607) [4]. Few non-compliant feeds exceeding authorized levels have been found during these years, with a maximum deteced concentration of 180 mg EQ/kg (224 mg ∑EQ+EQDM/kg) [4]. However, the presence of EQ in feed may result in an accumulation of both EQ and its breakdown products in the edible parts of farmed animals [5,6]. In several farmed fish species including, Atlantic cod (*Gadus morhua*), Atlantic salmon (*Salmo salar*), Atlantic halibut (*Hippoglossus hippoglossus*) and brown trout (*Salmo trutta*), the presence of EQ and its metabolites was confirmed [7]. In the EU, the authorization of EQ as a feed additive in the food production chain is currently under re-evaluation, and has meanwhile been formally suspended, because of a substantial lack of relevant toxicological data required for a risk assessment [8,9].

Several studies have indicated undesirable health-effects in animals fed diets containing EQ [10]. Yet, to date limited information is available on possible effects and effect levels of EQ present in fish feed [8]. In the large yellow croaker (*Pseudosciaena crocea*), ten weeks dietary exposure to 1350 mg EQ/kg feed decreased specific growth rate, condition factor and hepatosomatic index [11]. Furthermore, indications of perturbation of the lipid metabolism were seen at whole fish level with a curvilinear dose-response of crude lipid content to increasing concentrations of EQ. In turbot (*Scophthalmus maximus* L.), disturbed iono-regulatory mechanisms were suggested in fish exposed for 16 days to 200 and 400 mg EQ/kg feed, affecting plasma concentrations of sodium, chloride and calcium [12]. In tilapia (*Oreochromis niloticus*), 30 days of dietary exposure to 150 mg EQ/kg feed was found to have immunosuppressive effects and cause histopathological changes in the liver [13]. In Atlantic salmon (*Salmo salar* L.), EQ was shown to affect gene and protein expression patterns associated with the hepatic biotransformation of EQ through Phase I and II catalytic enzymes such as CYP1A1, CYP3A, GST and UDPGT after 90 days exposure to doses of up to 1800 mg/kg feed. However, no differences were seen in growth rate, feed conversion rate, hepato-somatic index or mortality [5,14].

In light of the scarce and conflicting data on EQ toxicity in farmed fish, the aim of the present study was to comprehensively investigate the toxicity and mode of action of subchronic dietary exposure to EQ in Atlantic salmon, using a systems biological approach. Five graded doses of EQ were administered to salmon through the diet and effects on health status were screened in liver, kidney, spleen and plasma. As a primary screening, metabolomic and proteomic profiling were performed and integrated in order to explore possible treatment-related changes and the interactions between different interconnected metabolic pathways. Identified target pathways were subsequently investigated by more targeted analyses using traditional physiological and biochemical measures to consolidate if these candidate pathways were of significance for the development of adverse outcomes. In order to characterize the risk from the dietary intake of EQ in Atlantic salmon, experimental data of toxicological responses were analyzed using benchmark dose models (BMD). The dose threshold for a toxic effect [15] was determined from the BMD of the critical endpoint of the study from which a safe upper level of intake for EQ for Atlantic salmon was proposed.

## Materials and methods

### Ethical statement

The experiment was approved by the Norwegian National Animal Research Authority (Mattilsynet; FOTS ID: 9004) and was performed in compliance with national and international ethical standards.

### Diet preparation

The experiment was designed as a subchronic oral toxicity study according to [16], including six dietary treatments. EQ-free diets were produced in one batch as 5 mm extruded pellets by EWOS Innovation (Bergen, Norway), according to an established recipe (Harmony Debio, organic fish feed; Table 1). The levels of EQ were adjusted by dissolving commercially available EQ (Capsoquin batch no. S-5162, 99% purity; courtesy of Industrial Técnica Pecuaria, S.A., Spain) directly into oil with subsequent coating of the pellets, aiming to obtain nominal EQ concentrations of 0, 50, 150, 1500, 5000 and 10 000 mg EQ/kg feed. Uncoated feed pellets with a lipid content 20% below the target level were mixed with EQ-containing fish oil and exposed to a reduced pressure of 0.1 bar for 10 min. The resulting oil-coated pellets were stored at -20°C throughout the experimental period. Concentrations of EQ and its main metabolite EQDM were measured in samples from each feed batch taken immediately after production (Table 2) and again after the 90 days feeding experiment. Ethoxyquin concentrations in feed were 0.47 mg (EQ 0), 41 ± 2 mg (EQ 1), 119 ± 7 mg (EQ 2), 1173 ± 113 mg (EQ 3), 3985 ± 228 mg (EQ 4) and 9666 ± 979 mg/kg feed at trial start, and did not show any degradation during the experimental period. Concentrations of EQDM were below the limit of quantification (LOQ <0.07 mg EQDM/kg feed) at both time points. To ensure nutritive stability of the control feed containing no added EQ, Vitamin E (500 mg/kg) and C (1 g/kg) were added as antioxidants to all diets. Analyses of the experimental diets confirmed equality of the proximate dietary composition between the different feeds. Measured levels of TBARS and vitamin E in the experimental diets were comparable (Table 2), and indicated oxidative stability of the diets throughout the experimental period.

**Table 1:** Formulation and proximate composition of 5 mm fish feed pellets spiked with graded levels of ethoxyquin (EQ) and fed to Atlantic salmon (*Salmo salar* L.) for 90 days.

Ingredients	Feed composition (g/ kg d.w.)
Fishmeal (EQ-free)^a^	219.7
Plant proteins^b^	327.8
Fish oil^c^	163.5
Rapeseed oil^d^	98.1
Wheat^e^	104.0
Fish protein concentrate^f^	60.0
Microingredients^g^	26.9
*Total*	*1000*.*0*
———Proximate analyses (% ww)^1^ ———	
Crude protein (N*6.25)	48.3 ± 2.4
Lipid	25.3 ± 1.8
Ash	5.8 ± 0.1
Dry matter	93.7 ± 0.5

^a^ Organic fish meal Norsildmel, Norway.

^b^ Soy protein concentrate, pea protein concentrate, wheat gluten.

^c^ Northern hemisphere fish oil.

^d^ Commodity, European origin.

^e^ Commodity, European origin.

^f^ Aquarius AS, Norway.

^g^ Vitamin E 50 (DL-alpha tocopherol), vitamin C 35% (mono-phosphate ascorbic acid). The vitamin and mineral mixture was prepared in order to meet the nutritional requirements of salmon as established by the National Research Council (2011).

^1^ Feed analyses were performed in technical duplicates. Given values represent the mean ± standard deviation of 6 parallel measurements.

**Table 2:** Concentrations of ethoxyquin (EQ), vitamin E and TBARS in the experimental diets fed to Atlantic salmon (*Salmo salar* L.) for 90 days.

	EQ (mg/kg ww)		Vitamin E (mg/kg ww)		TBARS (nmol/g ww)
Experimental diet	*Intended*	*Analyzed*	*α-tocopherol*	*γ-tocopherol*	
EQ 0	0	0.47	560	45	11.0
EQ 1	50	41 ± 2	670	47	12.0
EQ 2	150	119 ± 7	540	43	10.0
EQ 3	1500	1173 ± 113	650	44	9.8
EQ 4	5000	3985 ± 228	550	42	8.8
EQ 5	10000	9666 ± 979	570	40	9.2

EQ measurements were performed in technical duplicate. Given analyzed values represent the mean of one measurement in technical duplicate (EQ 0) or the means ± standard deviation of 4 parallel measurements performed in technical duplicates (EQ 1–EQ 5).

### Experimental conditions and sampling

The feeding trial was carried out at NOFIMA (Sunndalsøra, Norway) between July and October 2016. A total of 1260 individuals of 6 months old Atlantic salmon smolt (*Salmo salar*, L.) with an average initial weight of 150–200 g were randomly distributed into 18 tanks with 70 fish in each tank. All fish were sorted from the same group and shared the same genetic and environmental background. In order to maintain group homogeneity, fish deviating from the target size (± 15%) were discarded.

Fish were kept in indoor flow-through tanks with a surface area of 1.4 m^2^ and approximately 840 l water volume, under a photoperiod regime with 24h light. Water temperature, pH, oxygen availability and salinity were monitored daily during the experiment. Prior to trial start, the fish were acclimatized in their tanks on control feed containing no added EQ for a period of 14 days. Subsequently, three tanks were randomly assigned to each of the six experimental groups, and the fish were fed the experimental feeds for 90 days. The feeding regime was based on automatic feeders. Six daily meals were provided with four hours between the meals. Unconsumed feed pellets were collected and weighed once per day, and feed intake, feed conversion and EQ exposure were calculated.

Ten fish per tank (*n* = 30 per treatment group) were collected after both 45 and 90 days of exposure for tissue sampling. In addition, five fish per tank from the dietary group receiving an EQ concentration of ~150 mg/kg feed (EQ 2) were sampled at T = 0, and after 2, 4, 10, 20, 45 and 90 days of exposure, in order to monitor accumulation kinetics in fish fillet (results not reported here). In order to ensure equal treatment of all tanks, the number of fish in the other treatment groups was adjusted accordingly.

During sampling, fish were randomly collected from the tanks, anesthetized in a bath of tricaine methanesulfonate (FINQUEL MS-222; ~ 60mg L^-1^). Fish were sacrificed by a blow on the head and body weight and length of each fish were recorded. Five fish of each tank were homogenized to one pooled sample per tank for analysis of proximate composition. Of the five remaining fish, blood samples were taken from the caudal vein quickly following the initial anesthetization, using a heparinized VACUETTE blood collection tube with 21G x 1’ needle and kept on ice until further analyses and plasma separation. Liver, kidney, heart and spleen were collected and organ weights were recorded. Defined pieces of liver, kidney and spleen were fixed in 4% (v/v) formaldehyde (in PBS) for histopathological evaluation, while the rest of the tissue was immediately frozen in liquid nitrogen and kept at -80°C until further analysis. Individual muscle samples (Norwegian quality cut; NQC) were taken, homogenized, frozen and stored at -80°C.

### Calculations

In order to assess growth performance and EQ exposure of Atlantic salmon during the 90 days feeding trial, body weight gain, condition factor, specific growth rate, feed intake and feed conversion rate were calculated with the following equations:

Bodyweightgain=Finalbodyweight(g)−Meaninitialbodyweight(g) (1)

$$Condition\;factor\;(k)=\left(\frac{Final\;body\;weight\;(g)}{{Final\;body\;length\;(cm)}^{3}}\right)*100$$ (2)

$$Specific\;growth\;rate=\left(\frac{ln(Final\;body\;weight\;(g))-ln(Mean\;initial\;body\;weight(g))}{90\;days\;of\;feeding\;experiment}\right)*100$$ (3)

$$Daily\;feed\;intake*{fish}^{-1}=\frac{Recorded\;feed\;intake*{tank}^{-1}*{day}^{-1}(g)}{Number\;of\;fish*{tank}^{-1}}$$ (4)

$$Total\;feed\;intake*fish^{-1}=\sum Daily\;feed\;intake*fish^{-1}(g)$$ (5)

$$Feed\;conversion\;rate=\left(\frac{Total\;feed\;intake*{fish}^{-1}(g)}{Body\;weight\;gain\;(g)}\right)$$ (6)

$$Estimated\;daily\;EQ\;exposure=\left(\frac{\left(Daily\;feed\;intake*{fish}^{-1}(g))*Concentration\;of\;EQ\;in\;feed\;(mg/kg\right)}{Interpolated\;body\;weight\;(kg)}\right)$$ (7)

Organ somatic indices were calculated as the ratio of organ- to body weight.

### Quantification of ethoxyquin (EQ) and ethoxyquin dimer (EQDM)

The concentrations of EQ and EQDM in salmon muscle, whole body homogenates of five fish per tank (*n* = 3 tanks/group) and in salmon feed were performed as previously described by Bohne et al. [14], with modifications described by Ørnsrud et al. [17]. Briefly, EQ and EQDM were extracted with hexane from five individual muscle and five whole body homogenate samples per tank after saponification in ethanol-NaOH, or directly with 0.1% (w/v) solid acetic acid in acetonitrile from feed samples. Subsequently, the contents of EQ and EQDM were quantified by reversed-phase high-performance liquid chromatography (HPLC) with fluorescence detection, using an external standard curve.

### Proximate composition of diets and whole fish homogenates

Nitrogen content in feed samples and whole fish homogenates of five fish per tank (*n* = 3 tanks/group) was determined using a Vario Macro Cube (VMC) nitrogen analyzer according to AOAC [18], and crude protein was calculated as N*6.25. Total lipid content in feed samples was measured gravimetrically after acid hydrolysis or extraction with ethyl acetate from fish samples. Ash and dry matter were measured gravimetrically after ashing at 550 ± 5°C overnight or freeze drying for 48h, respectively.

### Vitamin E measurement

Vitamin E was extracted with hexane from feed and liver samples of five fish per tank (*n* = 3 tanks/group), after saponification with ethanol/ KOH. Tocopherol and tocotrienol isomers were quantified by HPLC with fluorescence detection, using an external standard curve as described by Lie et al. [19].

### Thiobarbituric acid reactive substances (TBARS)

Lipid peroxidation in feed and liver samples of five fish per tank (*n* = 3 tanks/group) was assessed through measurement of TBARS. The concentrations of TBARS were determined spectrophotometrically as described by Hamre et al. [20].

### Liver metabolomic profiling

In order to identify possible effects of dietary EQ exposure on liver metabolism, global metabolite profiles were determined in livers of Atlantic salmon from all dietary treatments (three randomly chosen fish per tank, 45 fish total) after 90 days of feeding. Metabolite profiling was performed by Metabolon Inc., USA, according to Metabolon’s standard methods. Briefly, samples were extracted and prepared for analysis, using the automated MicroLab STAR system (Hamilton Company, NV, USA). To remove protein, dissociate small molecules bound to protein or trapped in the precipitated protein matrix, and to recover chemically diverse metabolites, proteins were precipitated with methanol under vigorous shaking for 2 min (Glen Mills GenoGrinder 2000) followed by centrifugation. The sample extracts were then divided into four fractions. The organic solvent was removed, briefly placing the samples on a TurboVap (Zymark), the sample extracts were stored over night under nitrogen. All samples were analyzed using a combination of reverse phase (RP) ultra performance liquid chromatography mass spectrometry (UPLC-MS/MS) with positive and negative ion mode electrospray ionization (ESI), and hydrophilic interaction (HILIC) UPLC-MS/MS with negative ion mode ESI along with several internal standards. In preparation for the analyses, the sample extracts were dried and then reconstituted in solvents compatible to each of four methods. To quantify more hydrophilic compounds, one aliquot was analyzed using acidic positive ion conditions, gradient eluting the extract from a C18 column (Waters UPLC BEH C18-2.1x100 mm, 1.7 μm) using water and methanol, containing 0.05% perfluoropentanoic acid (PFPA) and 0.1% formic acid (FA). Another aliquot was also analyzed using acidic positive ion conditions, but chromatographically optimized for more hydrophobic compounds. For this method, the extract was gradient eluted from the same C18 column using methanol, acetonitrile, water, 0.05% PFPA and 0.01% FA and was operated at an overall higher organic content. The third aliquot was analyzed using basic negative ion optimized conditions, gradient eluting the basic extracts using a separate dedicated C18 column using methanol and water, however with 6.5mM Ammonium Bicarbonate at pH 8. The fourth aliquot was analyzed via negative ionization following elution from a HILIC column (Waters UPLC BEH Amide 2.1x150 mm, 1.7 μm) using a gradient consisting of water and acetonitrile with 10mM Ammonium Formate at a pH of 10.8. The MS analysis alternated between MS and data-dependent MSn scans using dynamic exclusion. All methods utilized a Waters ACQUITY UPLC and a Thermo Scientific Q-Exactive high resolution/accurate mass spectrometer coupled to a heated electrospray ionization source and an Orbitrap mass analyzer operated at 35 000 mass resolution.

Instrument variability was 5% for internal standards and total process variability for endogenous metabolites was 10%. Known compounds were identified by comparison to metabolomics library entries of purified standards.

### Liver proteomic profiling

Protein extraction from livers and tryptic protein digestions were performed on one randomly chosen fish per tank (*n* = 3/group) from the EQ 0, EQ 1, EQ 2 and EQ 3 groups at the Proteomics Unit at the University of Bergen, Norway (PROBE), according to in-house standardized protocols. In short, liver tissue samples were lyzed by sonication in 10μl lysis buffer/mg tissue (4% SDS, 0.1M Tris-HCl pH 7.6), using an ultrasonication rod (Q55 Sonicator, Qsonica, CT, USA) at 30% amplitude for 30 sec, or until tissue was dissolved. The lysed tissue was then incubated at 95°C for 7 min, and centrifuged for 10 min at 13000 rpm. The supernatant was collected and protein concentration determined Pierce BCA Protein assay kit (Thermo Scientific). 1M DiThiotreitol was added to the lysates, to obtain a final concentration of 0.1M, and the mix was incubated at 95°C for 5 min. The samples were further processed using a Filter Aided Sample Preparation (FASP) protocol with trypsin digestion as described by Wiśniewski et al. [21].

For Orbitrap Elite data-dependent-acquisition (DDA) analysis, between 0.5 and 1 ug protein, dissolved as tryptic peptides in 2% acetonitrile (ACN), 0.1% formic acid (FA), were injected into an Ultimate 3000 RSLC system (Thermo Scientific, Sunnyvale, California, USA) connected online to a linear quadrupole ion trap-orbitrap (LTQ-Orbitrap Elite) mass spectrometer (Thermo Scientific, Bremen, Germany), which in turn was equipped with a nanospray Flex ion source (Thermo Scientific). The sample was loaded and desalted on a pre-column (Acclaim PepMap 100, 2cm x 75μm ID nanoViper column, packed with 3μm C18 beads) at a flow rate of 5μl/min for 5 minutes (min) with 0.1% trifluoroacetic acid (TFA). Peptides were separated during a biphasic ACN gradient from two nanoflow UPLC pumps (flow rate of 270 nl /min) on a 50 cm analytical column (Acclaim PepMap 100, 50 cm x 75μm ID nanoViper column, packed with 3 μm C18 beads). Solvent A and B was 0.1% TFA (vol/vol) in water and 100% ACN, respectively. The gradient composition was 5% B during trapping (5min) followed by 5–7% B (over 1min), 7–21% B (134min), 21–34% B (45min), and 34–80% B (10min). Elution of very hydrophobic peptides and conditioning of the column were performed during 20 minutes isocratic elution with 80% B and 20 minutes isocratic elution with 5% B, respectively.

The eluting peptides from the LC-column were ionized in the electrospray and analyzed by the LTQ-Orbitrap Elite. The mass spectrometer was operated in DDA-mode to automatically switch between full scan MS and MS/MS acquisition. Instrument control was through Tune 2.7.0, and Xcalibur 2.2. Survey full scan MS spectra (from m/z 300 to 2,000) were acquired in the Orbitrap with a resolution of R = 240,000 at m/z 400 (after accumulation to a target value of 1e6 in the linear ion trap with maximum allowed ion accumulation time of 300ms). The 12 most intense eluting peptides above an ion threshold value of 3000 counts, and charge states of two or higher, were sequentially isolated to a target value of 1e4 and fragmented in the high-pressure linear ion trap by low-energy CID (collision-induced-dissociation) with normalized collision energy of 35% and wideband-activation enabled. The maximum allowed accumulation time for CID was 150 ms, the isolation with maintained at 2 Da, activation q = 0.25, and activation time of 10 ms. The resulting fragment ions were scanned out in the low-pressure ion trap at normal scan rate, and recorded with the secondary electron multipliers. One MS/MS spectrum of a precursor mass was allowed before dynamic exclusion for 40s. Lock-mass internal calibration was not enabled.

Prior to statistical analysis, the proteomics data were further processed using MaxQuant (version 1.6.0.1) and Perseus (version 1.6.0.2) as described in Tyanova et al. [22,23]. In short, MaxQuant running the built-in search engine Andromeda and protein sequences of the complete *Salmo salar* (Atlantic salmon) reference proteome downloaded from Uniprot (http://www.uniprot.org/proteomes/UP000087266) were used for protein identification and quantification [24]. For protein identification, carbamidomethylation of cysteines and protein N-terminal acetylation as well as oxidation of methionines were set as fixed modification and variable modification, respectively. Precursor mass tolerance was set to 4.5 ppm and 20 ppm were used for fragment ion identification. Up to two missed cleavages were allowed for trypsin digestion. Within MaxQuant, the software option “Match between runs” was enabled. The false discovery rates (FDR) for peptide and protein identifications were set to 1%. Only unique peptides were used for label-free quantification (LFQ). The MaxQuant output file was loaded into Perseus. Proteins identified only by site and reverse hits were removed from the dataset. Subsequently, the data was log_2_ transformed and the samples were grouped in to their respective categories. Relevant protein expression data including LFQ intensities, molecular weight, fold changes, statistical significance and protein accession numbers for protein identification are provided in S6 Table.

### Hematology

Hematocrit (Hct) was determined immediately from sampled blood of five fish per tank (*n* = 3 tanks/group) using Vitex Pari microhematocrit capillary tubes (Vitrex Medical A/S, Denmark) and a microhematocrit centrifuge (Haematofuge, Heraeus-Christ GmbH, Germany). The number of red blood cells (RBC) and amount of hemoglobin (Hb) in full blood were measured in a Cell Dyn 400 Hematological Analyzer (Sequoia- Turner) according to the manufacturer’s instructions, using Para 12 Extend control blood (Streck, MedMark Ref:218777) for calibration. Mean corpuscular volume (MCV), mean corpuscular hemoglobin (MCH) and mean corpuscular hemoglobin concentration (MCHC) were calculated from Hct, RBC and Hb as described in Sandnes et al. [25]. For a differential white blood cell count, blood smears were prepared on glass slides from a drop of heparinized blood. The smears were air dried, fixed in methanol for 5 min at room temperature, and stained with 50% May-Grünwald (Merck, Germany) and 10% Giemsa (Merck, Germany). The slides were examined in a light microscope using 60x and 100x magnifications, and a total of 100 leucocytes were counted and classified as neutrophils, lymphocytes or monocytes.

### Plasma biochemistry

Blood samples of five fish per tank (*n* = 3 tanks/group) were centrifuged at 3500*g* for 10 min to obtain the plasma fraction. The plasma was separated into aliquots, snap-frozen in liquid nitrogen and stored at -80°C until further analysis. Plasma concentrations of albumin and total protein, alanine aminotransferase (ALT), aspartate aminotransferase (AST), bile acids, bilirubin, creatinine and lysozyme were measured on a PL multipurpose diagnostic analyzer (Maxmat S.A., Montpellier, France) using DIALAB diagnostic kits (Vienna, Austria).

Osmolality was assessed by freezing point determination, using a Fiske One-Ten osmometer (Fiske, VT, USA). Sodium, potassium, chloride and free calcium in plasma were determined using the Radiometer ABL-77 Blood gas and electrolyte analyzer (Radiometer, Copenhagen, Denmark).

### Liver redox-homeostasis

In order to assess oxidative stress, the concentrations of reduced and oxidized glutathione (GSH and GSSG, respectively) in the liver were measured. Frozen liver tissue samples of five fish per tank (*n* = 3 tanks/group) were weighed and homogenized in either four times the volume of ice-cold 0.9% (w/v) saline buffer (9 g/L NaCl in ddH_2_O) for GSH analyses, or two times the volume of ice-cold thiol scavenger (*N*-ethylmaleimide pyridine derivative solution, Cat. No. GT35c; Oxford Biomedical Research, MI, USA) diluted 3:7 in 0.9% (w/v) saline buffer for GSSG analyses, using a ball mill (25 rpm for 1–2 min; Retsch MM301 ball mill, Haan, Germany). The homogenates were then centrifuged (5 min, 1500*g*, 4°C), and the supernatant was transferred to new tubes. The samples were further prepared using the Cuvette Assay kit for GSH/GSSG (Cat. No. GT35; Oxford Biomedical Research, MI, USA) following the manufacturer’s instructions, and GSH and GSSG were analyzed spectrophotometrically for absorbance at 405 nm in a Wallac VICTOR X5 2030 Multilabel Reader (PerkinElmer Life Sciences, MA, USA).

### Oxidative DNA damage

For assessment of oxidative DNA damage, genomic DNA was extracted from liver tissue using the AllPrep DNA/RNA Mini Kit (Qiagen, Germany). The formation of 8-hydroxy-2’-deoxyguanosine (8-oxo-dG) and apurinic/apyrimidinic (AP) sites was quantified in pooled DNA samples of five fish per tank (*n* = 3 tanks/group) using the commercial kits HT 8-oxo-dG ELISA kit II (Trevigen Inc., MD, USA) and OxiSelect Oxidative DNA Damage Quantitation Kit (AP sites) (both Cell Biolabs INC., CA, USA), following the manufacturer’s instructions. Results are expressed as nM 8-oxo-dG per unit DNA (ug/ul) and AP sites per 10^5^ base pairs (bp), respectively.

### Histology

Tissue samples of liver, spleen and kidney of five fish per tank were fixed in 4% (v/v) formaldehyde overnight, washed in PBS and then stored in 70% (v/v) ethanol until further processing. The fixed tissues were further dehydrated through graded alcohols and xylene, and finally embedded in paraffin. Tissue sections of 5 μm were then stained with hematoxylin and eosin (H&E) and periodic acid-Schiff stain (PAS) for histopathological evaluation. In addition, the special staining Pearl’s Prussian blue was employed to evaluate the presence of iron-derived pigments. Sections were scanned with a ZEISS Axio Scan.Z1 (Carl Zeiss A/S, Birkeroed, Denmark). The histopathological changes were evaluated in each tissue based on the spectrum of lesions, and presence was graded giving scores from 0–2 or 0–3. All analyses were performed in a double-blinded format.

### Statistical and bioinformatic analyses

Statistical data analysis was performed in R, version 3.4.0 [26]. Measurements performed on tank pooled samples or tank means (*n* = 3/group) were analyzed with one-way ANOVA and Tukey’s test for multiple comparison of group means. Normal distribution of the model residuals and homogeneity of variance amongst treatment groups were tested using the Shapiro-Wilk test and Levene’s *F*-test, respectively. Data not complying with the criteria of the ANOVA, were analyzed using Kruskal-Wallis test, and a Wilcoxon’s test for multiple pairwise comparison of the groups. For parameters including replicate measurements from the individual tanks, a nested ANOVA was fit using a mixed effects model with tank treated as a random effect. Normal distribution, bias and homoscedasticity of the model residuals were checked, and models were fitted on z-normalized data where necessary. Post-hoc group comparison was performed on least-square means adjusting for multiple comparisons.

Statistical analyses of data from histopathological evaluation were performed using IBM SPSS Statistics 22 (Released 2013; IBM Corp., NY, USA), employing Pearson’s X^2^ (level of confidence 95%) for pairwise comparison of group mean scores.

Results are presented as means ± SD of all measurements. Statistical significance compared to the control group (EQ 0) is denoted with * *p*<0.05.

Data processing and statistical comparison of liver global metabolomic and proteomic profiles was performed using the Qlucore Omics Explorer version 3.1 (Qlucore AB, Lund, Sweden). Original scale raw area counts of all biochemicals and proteins were mean centered and log_2_ transformed. The data were analyzed using one-way ANOVA followed by planned contrasts, comparing the liver metabolite and protein profiles of each exposure group to the livers of unexposed animals. For statistical analyses, a p-values of *p*<0.05 was used as significance cut-off and multiple test corrected *p*-values were calculated and reported. The data were further explored using unsupervised principal component analysis and hierarchical cluster analysis.

For a general biological pathway analysis of biochemical, Kyoto Encyclopedia of Genes and Genomes (KEGG) accession numbers were determined for metabolites whose abundances were significantly altered when compared to the control (EQ 0), and subsequently imported into the Ingenuity Pathway Analysis software suite (IPA; Quiagen, CA, USA). In order to interpret the proteomic data obtained from salmon livers, mammalian orthologs of *Salmo salar* UniProt accession numbers were determined and uploaded to IPA. Consistent with the metabolomics pathway analysis, only proteins significantly altered in comparison to EQ 0 were included into the analyses. IPA Core Analyses were performed on both successfully mapped metabolites and proteins using default settings. For a concomitant interpretation of the multi-omics dataset, an IPA Comparison Analysis was performed followed by targeted upstream analyses as described in Rasinger et al. [27].

### Benchmark dose assessment

Benchmark dose (BMD) analyses were conducted according to the benchmark dose technical guidance by the European Food Safety Authority (EFSA) [15,28]. For continuous data, two families of (nested) models; the exponential and Hill models, were fit on individual data using the EFSA BMD platform (Proast, version 64.16; https://shiny-efsa.openanalytics.eu/app/bmd). Ordinal data from histology were transformed into quantal data and the default set of models [28] was fit on individual data using the EFSA BMD software (Proast, version 61.3) locally run in R.

Selection of models was based on the Akaike information criterion (AIC). A default value of 2 units difference between AICs is considered as the critical value by the EFSA [28]. BMD models were accepted when the AIC of the model was lower than the AIC of the null model (no dose response) -2 (AIC<AICnull-2), and the model with lowest AIC (AICmin) was lower than the AIC of the full model +2 (AICmin<AICfull+2) [28]. As model averaging is recommended as the preferred method for calculating the BMD confidence interval, model averaging was performed for those data sets (quantal data: histology parameters) where this option was available in the current publicly version of Proast. For data sets were no averaging option was available (continuous data: plasma and blood parameters), the best model based on AIC was used as described by the EFSA [28]. For continuous nested data, two models (model 3 and 5) were considered from the exponential and hill model families, and from these the model with the lowest AIC was selected for calculating the BMD 90% confidence interval; the lower bound is reported as BMDL and the upper bound as BMDU. The confidence intervals for the BMD for nested models were estimated including bootstrap with standard 200 Bootstraps. The BMD is defined as the dose that corresponds with a specified estimated change in response compared with the modelled background response. The BMR (benchmark response) is the estimated response corresponding with the BMD of interest. A default of BMR of 5% change was used as starting point for analyses for continuous data (BMDL_05_). However, where a wide BMD confidence interval (BMDU/BMDL) indicated poor precision of the estimate, the BMR was increased as described in the EFSA technical guidance document [28], and the BDML for BMRs of 10 or 20% changes were considered (BMDL_10_ and BMDL_20_, respectively) for plasma enzymes or adaptive responses (biomarkers of exposure). For quantal and ordinal data (histology) the default BMR was defined as a specified increase in incidence over background, and BMR of 10% (extra risk; BMDL_10_) was used as described by the EFSA [28].

## Results

### Feed intake, growth and body composition

After 90 days of dietary exposure to graded levels of EQ no mortality or gross signs of toxic responses were observed in any of the experimental groups. No significant effects on feed intake and growth performance were observed at doses up to EQ 3. However, exposure to dietary levels above this level, i.e. EQ 4 and EQ 5, significantly (*p*<0.05) reduced feed consumption (Table 3). Hence, fish fed EQ 4 and EQ 5 displayed poor growth performance, as indicated by a feed intake dependent reduction in specific growth rate, condition factor, and final body weight when compared to fish from the control group (EQ 0). Concomitant to lower body weight, organ masses of heart, liver and spleen were significantly decreased in fish of the EQ 5 group, but not when normalized to body weight (cardio-, hepato- and spleenosomatic index, respectively).Thus, no treatment-related effects on organ weights were observed.

**Table 3:** Growth parameters, feed intake, exposure estimation, survival, proximate body composition and organ weights of Atlantic salmon (*Salmo salar* L.) exposed to graded levels of EQ through their diet for 90 days.

	Dietary treatment						
*Growth parameters*	EQ 0	EQ 1	EQ 2	EQ 3	EQ 4	EQ 5	P-values
Final length (cm)	36.3 ± 3.5	37.0 ± 3.6	37.6 ± 3.3	37.9 ± 3.0	34.4 ± 3.1	29.4 ± 3.2*	< .0001
Final body weight (g)	716.3 ± 234.5	728.1 ± 222.9	784.5 ± 197.2	756.7 ± 190.7	537.2 ± 155.5*	301.5 ± 111.5*	< .0001
Body weight gain (g)	454.2 ± 234.5	467.5 ± 222.4	515.3 ± 197.4	493.9 ± 190.6	275.8 ± 155.4*	37.61 ± 111.7*	< .0001
Specific growth rate (%*day^-1^)	1.1 ± 0.3	1.1 ± 0.3	1.2 ± 0.3	1.1 ± 0.3	0.8 ± 0.3*	0.1 ± 0.4*	< .0001
Condition factor (g/cm^3^)	1.5 ± 0.1	1.4 ± 0.1	1.4 ± 0.1	1.4 ± 0.1	1.3 ± 0.1*	1.1 ± 0.1*	< .0001
Mean daily feed intake (g*fish^-1^)^a^	4.9 ± 0.1	4.6 ± 0.6	5.1 ± 0.3	4.4 ± 0.4	2.9 ± 0.1*	1.1 ± 0.3*	< .0001
Mean total feed intake (g*fish^-1^)^b^	435.9 ± 4.0	407.4 ± 50.7	452.6 ± 25.5	397.6 ± 32.5	255.0 ± 6.2*	100.0 ± 24.4*	< .001
Feed conversion rate (*fish^-1^)	1.0 ± 0.2	0.9 ± 0.1	0.9 ± 0.1	0.8 ± 0.1	0.9 ± 0.1	4.0 ± 3.3	0.072
Total EQ consumed (mg*fish^-1^)	0.205 ± 0.002	17 ± 2	54 ± 3	466 ± 38*	1016 ± 25*	966 ± 236*	< .001
Estimated daily EQ exposure (mg*kg BW^-1^*day^-1^)^c^	0.0049 ± 0.0002	0.41 ± 0.05	1.22 ± 0.04	11 ± 1*	30 ± 2*	40 ± 10*	< .001
Survival (%)	100	100	100	100	100	100	
*Proximate body composition whole fish (% ww)*							
Crude protein (N*6.25)	17.3 ± 0.6	18.0 ± 0.0	17.7 ± 0.6	18.0 ± 1.0	17.7 ± 0.6	17.3 ± 0.6	0.643
Crude lipid	15.2 ± 0.4	14.1 ± 0.2	14.1 ± 0.5	13.3 ± 0.4*	11.0 ± 0.4*	8.7 ± 1.3*	< .001
Ash	1.8 ± 0.2	1.8 ± 0.2	2.0 ± 0.2	1.8 ± 0.1	1.9 ± 0.1	2.1 ± 0.1	0.207
Dry matter	34.0 ± 1.4	33.3 ± 0.7	33.9 ± 1.6	33.3 ± 0.6	30.0 ± 0.1*	27.8 ± 1.1*	< .001
Crude lipid muscle	4.1 ± 1.0	3.5 ± 0.1	3.7 ± 0.7	4.0 ± 0.4	2.7 ± 0.1*	1.8 ± 0.7*	< .01
*Organ weights*							
Heart (g)	0.8 ± 0.3	0.8 ± 0.2	0.9 ± 0.2	0.8 ± 0.2	0.6 ± 0.2	0.4 ± 0.1*	< .001
Cardiosomatic index (*100)	0.12 ± 0.01	0.10 ± 0.01	0.11 ± 0.01	0.10 ± 0.01	0.11 ± 0.01	0.12 ± 0.02	0.108
Liver (g)	8.0 ± 2.9	7.8 ± 2.4	8.8 ± 2.2	8.2 ± 2.5	6.7 ± 2.0	3.2 ± 1.3*	< .001
Hepatosomatic index (*100)	1.05 ± 0.10	1.02 ± 0.08	1.08 ± 0.08	1.08 ± 0.12	1.16 ± 0.10	1.01 ± 0.20	0.102
Spleen (g)	0.6 ± 0.2	0.6 ± 0.3	0.6 ± 0.2	0.6 ± 0.3	0.5 ± 0.1	0.2 ± 0.1*	< .01
Spleen somatic index (*100)	0.08 ± 0.02	0.08 ± 0.02	0.08 ± 0.01	0.08 ± 0.02	0.09 ± 0.03	0.07 ± 0.02	0.067

Measurements performed on tank pooled samples or tank means (*n* = 3/group) were analyzed performing one-way ANOVA and Tukey’s test for multiple comparison of group means. For parameters including replicate measurements from the individual tanks, a nested ANOVA was fit using a mixed effects model (*n* = 25–30) with tank treated as a random effect, and post-hoc comparison of least-square group means adjusting for multiple comparisons. Results are presented as means ± SD of all measurements.

* *p*<0.05, compared to the control (EQ 0).

^a^ Daily feed intake recorded per tank divided by number of fish.

^b^ Sum of daily feed intake per fish

^c^ Calculated from daily feed intake per fish and interpolated body weight per fish. Abbreviation: BW, Body weight.

The proximate body composition of the fish was assessed through measurement of crude protein, crude lipid, total ash and dry matter in whole fish, as well as the lipid content of the muscle. Total protein content of whole fish homogenates was between 17.3 ± 0.6 (EQ 0) and 18.0 ± 1.0 (EQ 3) % ww, and was not affected by dietary EQ exposure of up to 40.0 ± 9.6 mg/ kg BW/ day (EQ 5). Meanwhile, fish fed EQ 4 and EQ 5 had significantly (*p*<0.05) reduced levels of total body fat, total lipid content in muscle and total body dry matter, reflecting the lower growth rate. Although not affecting lipid content in the fillet, fish fed EQ 3 had an overall reduction in whole body fat, which could not be explained by reduced feed intake.

### Tissue accumulation of ethoxyquin (EQ) and ethoxyquin dimer (EQDM)

Despite the gradually reduced feed intake and growth in EQ 4 and EQ 5, there was a dose-dependent whole body accumulation of EQ and EQDM (S1 Table). The concentration of EQ relative to the concentration of EQDM increased with increasing EQ exposure in whole fish. Similarly, there was a dose-dependent increase of EQ and EQDM in muscle; however, the concentration of EQ relative to the concentration of EQDM peaked at EQ 3 and decreased for EQ 4 and EQ 5.

### Liver metabolomic profiling and pathway analysis

In a search for the underlying mode of action of the biological effects of EQ, an in depth metabolic screening was performed on liver tissue. A total of 498 named biochemicals were detected, of which 371 were significantly (*p*<0.05, ANOVA) affected by the treatment. Because of the potential bias resulting from the significantly reduced feed intake in animals exposed to doses above EQ 3, namely EQ 4 and EQ 5, were excluded from further analyses, yielding a total of 192 metabolites (38, 60 and 152 metabolites in EQ 1, EQ 2 and EQ 3, respectively, as compared to EQ 0), which were directly affected (*p*<0.05, ANOVA) by EQ treatment.

**Fig 1:**
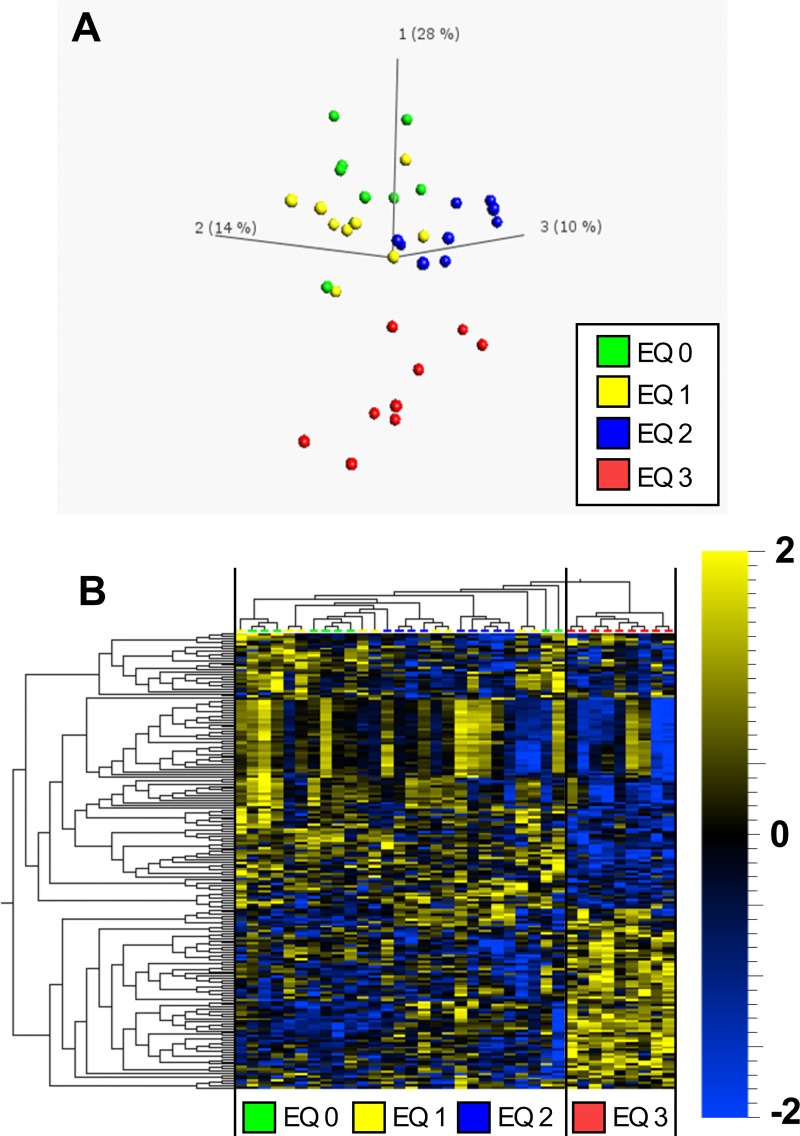
Principal component analysis (PCA) and hierarchical clustering analysis (HCA) of 192 significantly regulated (*p*<0.05, ANOVA) metabolites in livers of Atlantic salmon (*Salmo salar* L.) exposed to graded levels of ethoxyquin (EQ) through their diet for 90 days. Differential analysis (ANOVA), PCA **(A)** and HCA **(B)** were performed using the Qlucore omics-explorer. The heatmap (B) represents the concentrations of significantly affected metabolites within each measured sample (represented in the columns). Yellow bars indicate higher concentrations of a metabolite, while blue bars indicate lower concentrations of a metabolite, on a scale from -2 to 2, where 1 unit is equal to a variance of 1 from the mean. See S2 Table for a complete overview of individual metabolites.

Global liver metabolite profiles of fish exposed to graded levels of EQ were examined through unsupervised PCA and HCA. The overall percentage of explained variance of the first three components was low (52%). However, the PCA of liver metabolomic data revealed that the metabolite profile of fish exposed to doses including EQ 0, EQ 1 and EQ 2 formed an overlapping population that clearly separated from fish exposed to EQ 3 along component 1 (Fig 1A). HCA revealed separation based on the dose of EQ with control and lower doses of EQ (EQ 0, EQ 1 and EQ 2) grouped as metabolically similar (Fig 1B; left side of the panel). Liver metabolite profiles of fish exposed to EQ 3 formed a separate cluster (Fig 1B; right side of the panel).

Abundance of metabolites significantly altered compared to the control revealed metabolic responses following exposure to dietary EQ higher than EQ 2 with 85 metabolites that were only affected after exposure to EQ 3 (S4 Table). These responses were most notably associated with liver energy metabolism, redox- homeostasis and purine/pyrimidine metabolism.

#### Energy metabolism

**Fig 2:**
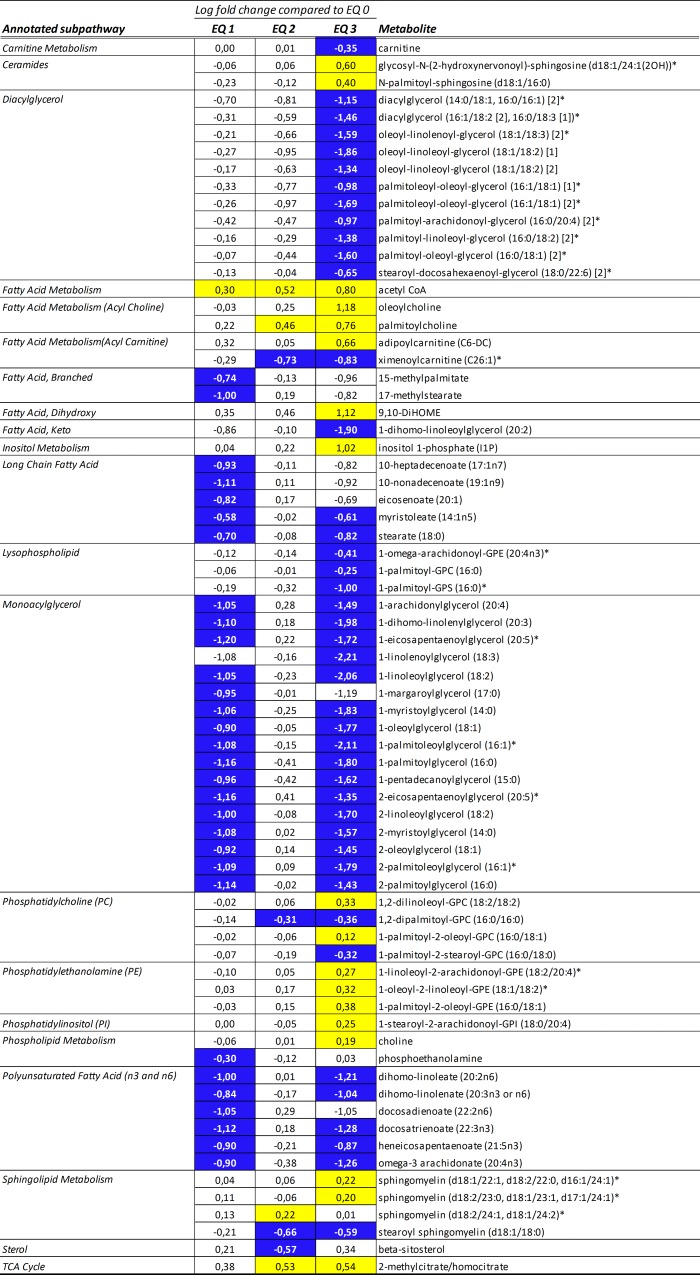
Heat map of significantly altered metabolites (ANOVA, *p*<0.05) belonging to the “lipid” super pathway in livers of Atlantic salmon (*Salmo salar* L.) exposed to graded levels of ethoxyquin (EQ) through their diet for 90 days. Scaled intensity means were used to calculate log2 fold changes in the individual exposure groups compared to unexposed control animals (EQ 0). Significance of change was analyzed through pairwise comparisons between the individual exposure groups and the control, with colors denoting a significantly increased (yellow) or decreased (blue) levels compared to the control. See S4 Table for a complete overview of individual metabolites.

The in-depth metabolic screening revealed marked changes in several pathways related to energy metabolism. Altered metabolite profiles related to hepatic lipid metabolism were characterized by a decrease in diacylglycerol (e.g., palmitoleoyl-oleoyl-glycerol (16:1/18:1)) and monoacylglycerol species (e.g., 1-palmitoleoylglycerol (16:1); Fig 2). Also, significantly lower levels of long-chain fatty acids (e.g., stearate (18:0)) and polyunsaturated fatty acids (e.g., omega-3 arachidonate (20:4n3)) were noted. A decrease in carnitine-, and concomitant increase in acetyl-CoA levels suggested increased beta-oxidation rates in livers of fish exposed to EQ 3 compared to EQ 0 (Fig 2).

**Fig 3:**
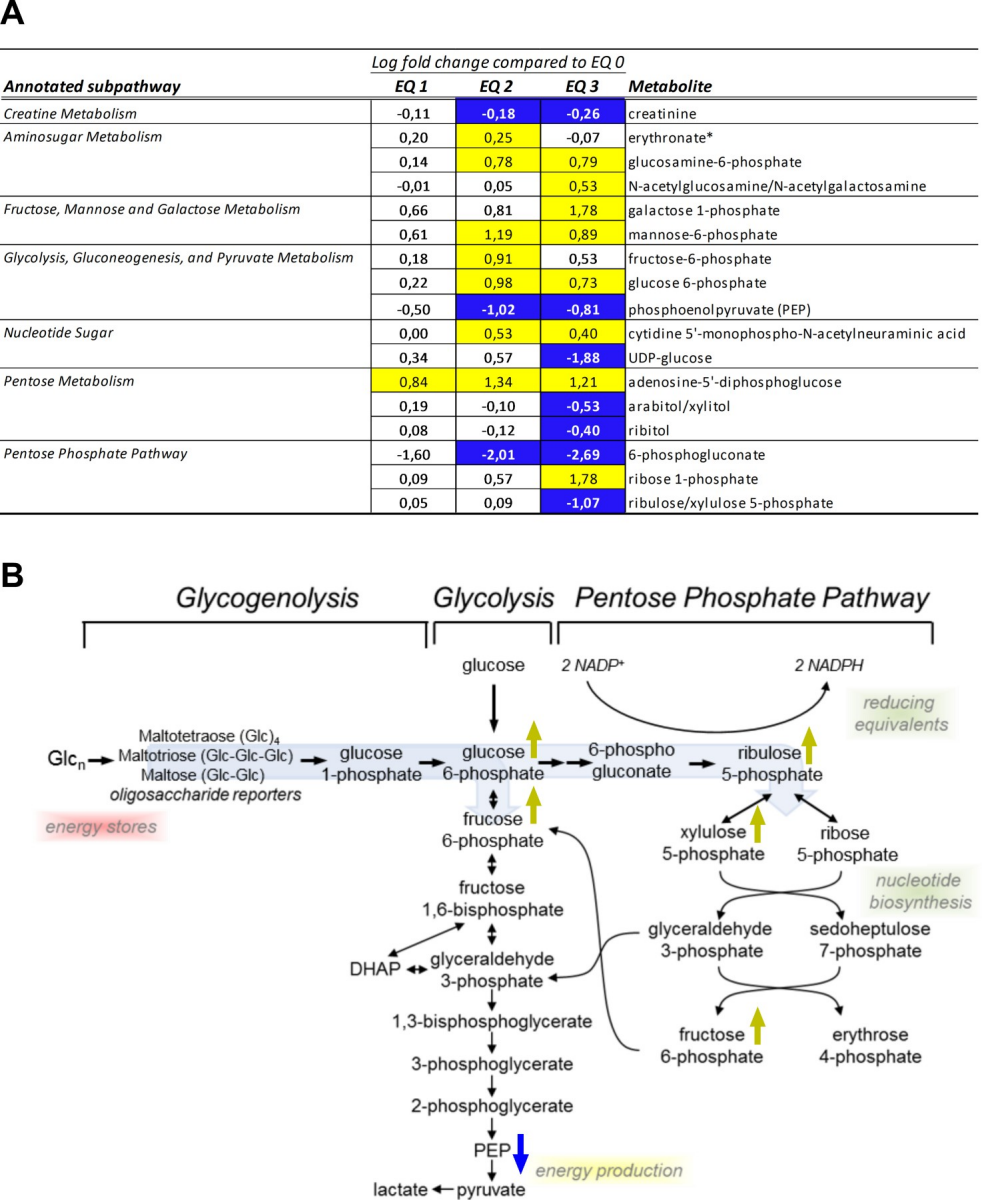
Alterations in liver creatinine levels and carbohydrate metabolism in livers of Atlantic salmon (*Salmo salar* L.) exposed to graded levels of ethoxyquin (EQ) through their diet for 90 days. **(A)** Metabolites displaying differential expression (*p*<0.05, ANOVA) were grouped according to subpathway annotations. Scaled intensity means were used to calculate log2 fold changes in the individual exposure groups compared to unexposed control animals (EQ 0). Significance of change was analyzed through pairwise comparisons between the individual exposure groups and the control, with colors denoting a significantly (*p*<0.05) increased (yellow) or decreased (blue) levels compared to the control. **(B)** Overview of selected significantly affected carbohydrate metabolites within the pathways of glycogenolysis, glycogenolysis and the pentose phosphate pathway. Metabolites significantly (*p*<0.05) affected by the increasing EQ levels are denoted with yellow (increase) or blue (decrease) arrows. See S4 Table for a complete overview of individual metabolites.

Furthermore, metabolic pathways involved in glucose metabolism and energy generation were affected by the exposure to EQ. EQ treatment increased the levels of glycolytic intermediates glucose 6-phosphate and fructose 6-phosphate (Fig 3A and 3B), and caused a dose-dependent decrease in hepatic creatinine (sig. >EQ 2; Fig 3A). In addition, EQ treatment resulted in altered levels of pentose phosphate pathway biochemicals: 6-phosphogluconate levels were decreased in a dose-dependent manner (significantly decreased following 90 days exposure to levels above EQ 1), while ribose-1-phosphate levels were elevated. At the same time, lower levels of the pentose sugars ribitol and arabitol/xylitol were observed with increasing concentrations of EQ.

#### Liver redox-homeostasis

**Fig 4:**
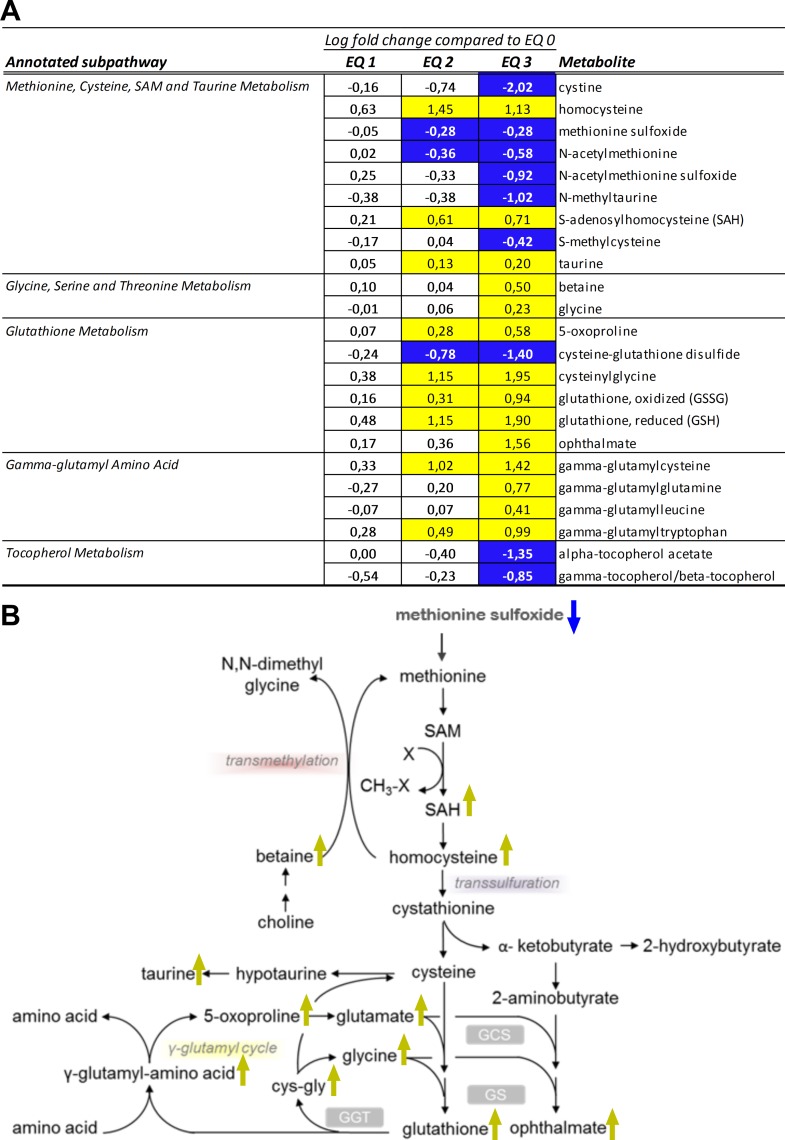
Changes in redox homeostasis in livers of Atlantic salmon (*Salmo salar* L.) exposed to graded levels of ethoxyquin (EQ) through their diet for 90 days. **(A)** Metabolites displaying differential expression (*p*<0.05, ANOVA) were grouped according to subpathway annotations. Scaled intensity means were used to calculate log2 fold changes in the individual exposure groups compared to unexposed control animals (EQ 0). Significance of change was analyzed through pairwise comparisons between the individual exposure groups and the control, with colors denoting a significantly (*p*<0.05) increased (yellow) or decreased (blue) levels compared to the control. **(B)** Overview of selected treatment affected metabolites within the cysteine and glutathione metabolic pathways. Metabolites significantly (*p*<0.05) affected by the increasing EQ levels are denoted with yellow (increase) or blue (decrease) arrows. See S4 Table for a complete overview of individual metabolites.

The glutathione system represents a major cellular redox buffer that significantly contributes to the maintenance of the reduced intracellular milieu and, hence, to the anti-oxidative capacity of cells. Ninety days of dietary exposure to graded levels of EQ caused a substantial increase in the total glutathione pool (both reduced and oxidized) in livers of Atlantic salmon. The levels of reduced glutathione (GSH) were elevated in a dose-dependent manner following EQ treatment and to the higher degree than the levels of oxidized species (GSSG) (Fig 4A). Cysteine, the limiting factor in GSH biosynthesis, is acquired by cells as cystine (cysteine disulfide) or synthesized from methionine through the transmethylation and transsulfuration pathways. Notably, while hepatic cysteine levels (S3 Table) were not affected by the EQ treatment, levels of cystine were significantly reduced (Fig 4A). Concomitantly, altered levels of metabolites involved in transmethylation and transsulfuration pathways (Fig 4B), including reduced levels of methionine precursors, as well as S-methylcysteine and N-methyltaurine (Fig 4A), and increase in homocysteine and S-adenosylhomocysteine (SAH) and taurine (Fig 4A), were suggestive of increased utilization of these substrates to support GSH biosynthesis. These changes were accompanied by an increase in gamma-glutamyl amino acids (e.g., gamma-glutamylcysteine, gamma-glutamylleucine), which are formed when the enzyme gamma-glutamyl transpeptidase (GGT) transfers the gamma-glutamyl moiety from glutathione to an acceptor amino acid, as well as a concentration-dependent increase in 5-oxoproline and cysteinylglycine (Fig 4A). The levels of ophthalmate were significantly increased following exposure to EQ 3, while both alpha- and gamma-tocopherol significantly decreased. Ophthalmate is an endogenous analogue of GSH, which is considered a marker of GSH consumption under oxidative stress conditions. Moreover, a dose-dependent elevation in the levels of dihydroxy fatty acid 9,10-DiHOME (Fig 2), significantly increased after exposure to EQ 3, was observed. As dihydroxy fatty acids are oxidation products derived from essential polyunsaturated fatty acids, elevated levels of DiHOME fatty acids, derived from linoleic acid (18:2n-6), are reflective of hepatic oxidative stress. Thus, the observed treatment-related changes in salmon liver metabolites reflected increased utilization of glutathione and a compensatory induction of redox capacity, indicating a perturbation of liver redox-homeostasis and presence of oxidative stress.

#### Purine and pyrimidine metabolism

**Fig 5:**
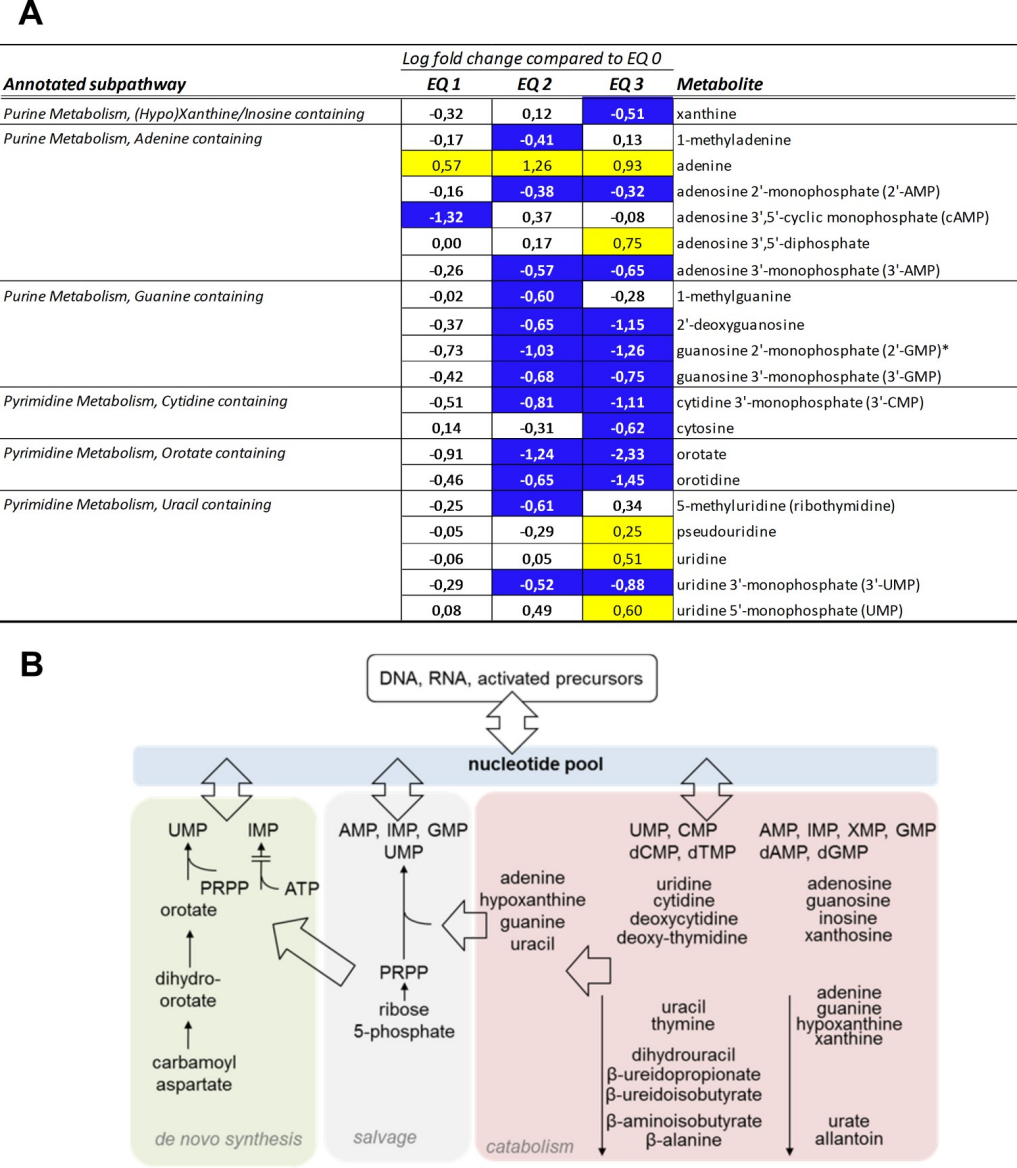
Alterations in purine and pyrimidine metabolism in livers of Atlantic salmon (*Salmo salar* L.) exposed to graded levels of ethoxyquin (EQ) through their diet for 90 days. **(A)** Metabolites displaying differential expression (*p*<0.05, ANOVA) were grouped according to subpathway annotations. Scaled intensity means were used to calculate log2 fold changes in the individual exposure groups compared to unexposed control animals (EQ 0). Significance of change was analyzed through pairwise comparisons between the individual exposure groups and the control, with colors denoting a significantly (*p*<0.05) increased (yellow) or decreased (blue) levels compared to the control. See S4 Table for a complete overview of individual metabolites. **(B)** Overview of nucleotide metabolites within the pathways of nucleotide synthesis, salvage, and catabolism.

Treatment related alterations in purine and pyrimidine metabolism were observed after dietary treatment to EQ. A number of purine and pyrimidine intermediates showed changes after exposure to increasing concentrations of EQ (Fig 5A). The concentration of purine and pyrimidine are dependent on the equilibrium between both *de novo* synthesis and release from salvage pathways, and the rate of catabolism (Fig 5B). The levels of adenine were higher when comparing samples from EQ-exposed animals to controls, and were detectable above exposure to EQ 0 (Fig 5A). Orotate and orotidine containing pyrimidines were decreased above exposure to EQ 1, guanine containing purines along with cytidine containing pyrimidines were increased above exposure to EQ 1, while uracil containing pyrimidines showed a less consistent response with both increased and decreased levels for the different compounds.

### Liver proteomic profiling

In order to further investigate potential molecular mechanisms underlying the observed effects of dietary EQ exposure on hepatic metabolites, proteomic profiling was performed on liver sections of fish (*n* = 3/group) fed the EQ 0, EQ 1, EQ 2 and EQ 3 diet, respectively. A total of 3394 proteins were detected, of which 287 were significantly (*p*<0.05, ANOVA) affected by the treatment. Post hoc analysis using planned contrasts revealed that EQ 1, EQ 2 and EQ 3 induced significant (*p*<0.05) changes in the abundance of 57, 80 and 164 proteins, respectively.

**Fig 6:**
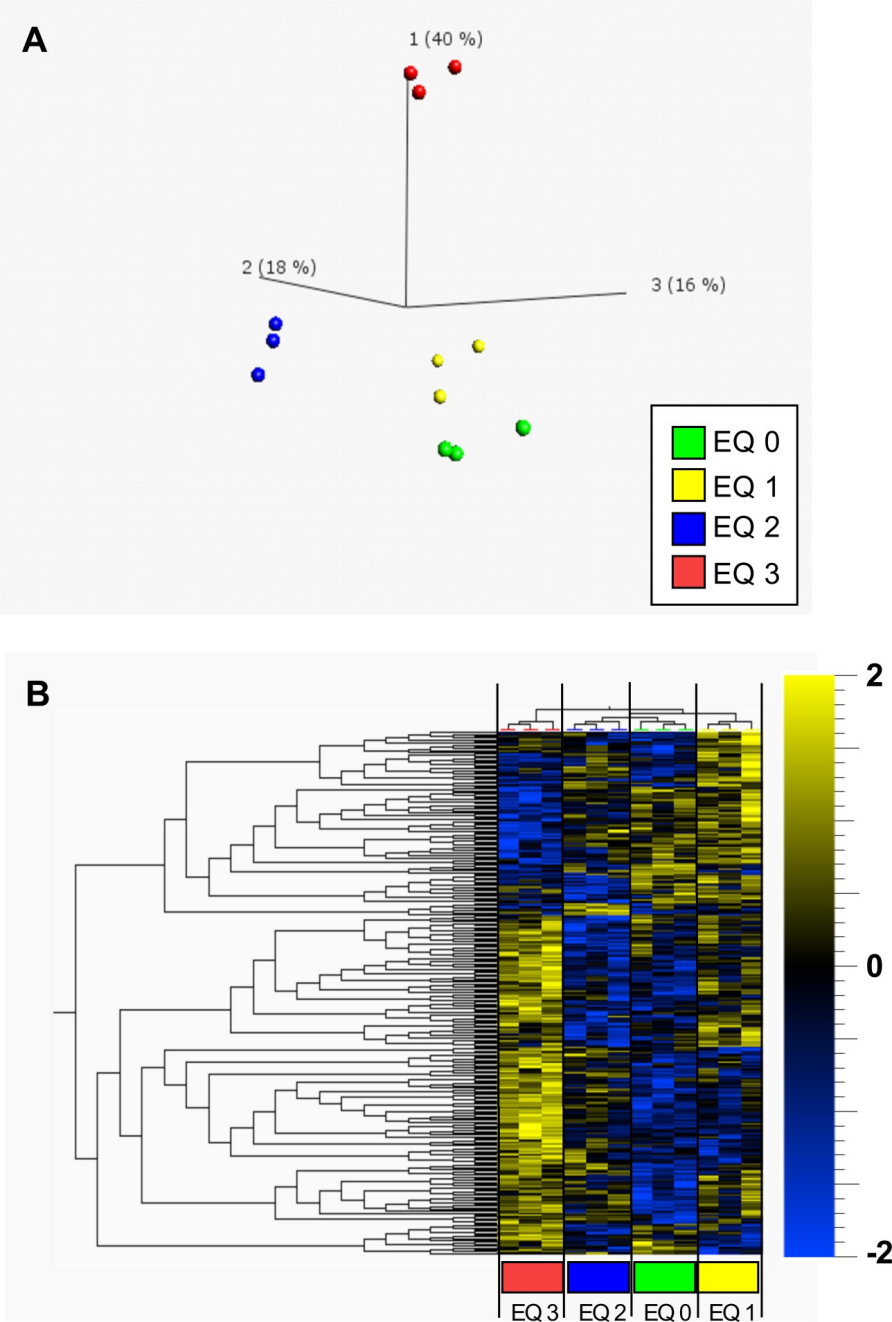
Principal component analysis (PCA) and hierarchical clustering (HC) of 287 significantly regulated (*p*<0.05, ANOVA) proteins in livers of Atlantic salmon (*Salmo salar* L.) exposed to graded levels of ethoxyquin (EQ) through their diet for 90 days. Differential analysis (ANOVA), PCA **(A)** and HC **(B)** were performed using the Qlucore omics-explorer. The heatmap (B) represents the concentrations of significantly regulated proteins within each measured sample (represented in the columns). Yellow bars indicate higher concentrations of a protein, while blue bars indicate lower concentrations of a protein, on a scale from -2 to 2, where 1 unit is equal to a variance of 1 from the mean. See S9 Table for a complete overview of individual proteins.

Global liver proteome profiles of fish exposed to graded levels of EQ were examined through unsupervised principal component analysis (PCA) and hierarchical clustering analysis (HCA). The overall percentage of explained variance of the first three components was 74% and in accordance with the metabolite data revealed a clear separation between EQ 3 and the exposure groups based on the lower doses of EQ (EQ 0, EQ 1 and EQ 2) along component 1 (Fig 6A).

HCA revealed a similar and more clearly resolved separation of the exposure groups based on the dose of EQ (Fig 6B). Liver protein profiles of fish exposed to EQ 3 formed a separate cluster from control and lower doses of EQ (EQ 0, EQ 1 and EQ 2). Unlike the metabolite data, the proteomic data also allowed for a clear distinction between EQ 0, EQ 1 and EQ 2.

**Fig 7:**
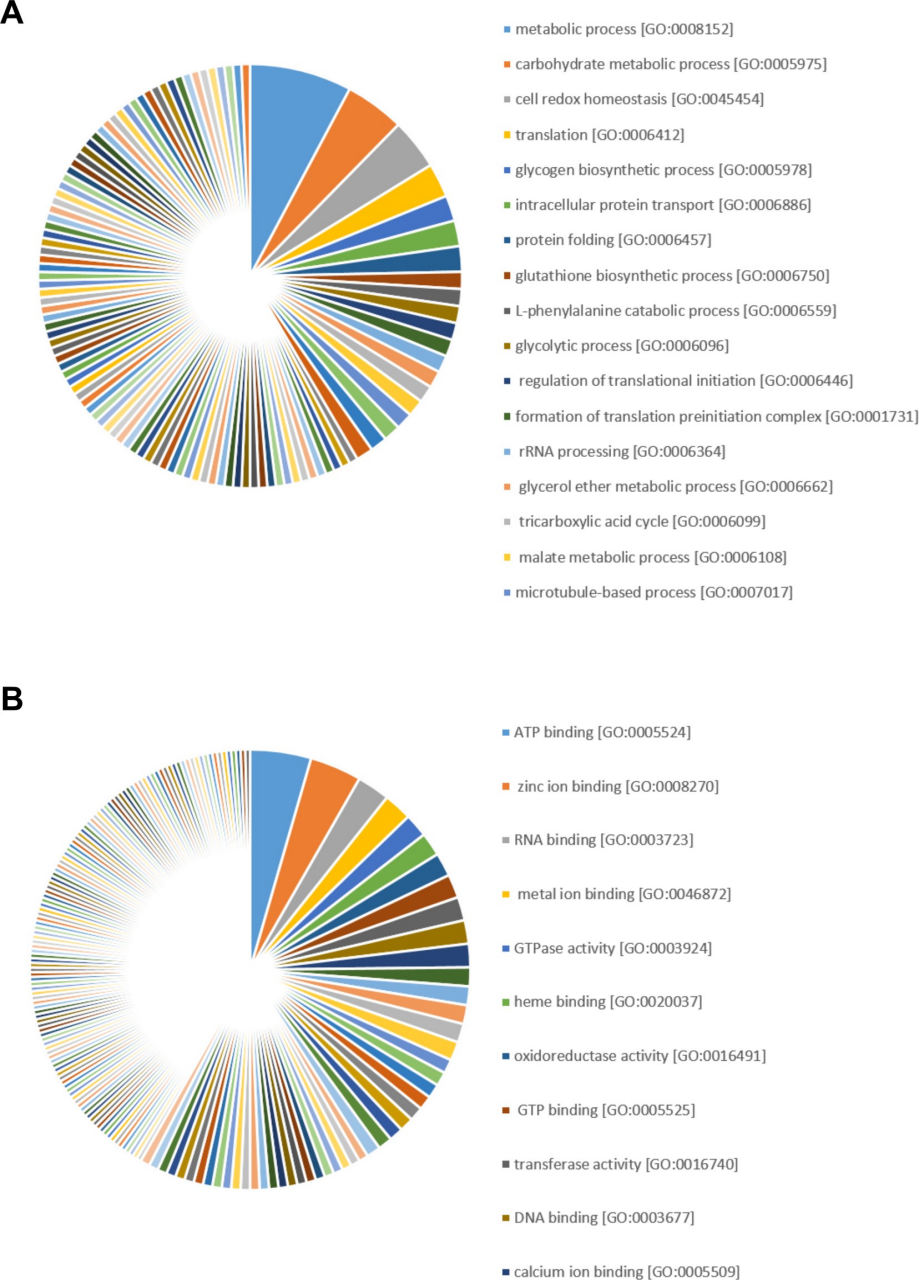
Gene ontology analysis of significantly regulated (*p*<0.05, ANOVA) proteins in livers of Atlantic salmon (*Salmo salar* L.) exposed to graded levels of ethoxyquin (EQ) through their diet for 90 days. Proteins displaying differential expression (*p*<0.05, ANOVA) were grouped into the gene ontology categories biological processes **(A)** and molecular functions **(B)**. See S7 Table for a complete overview of individual proteins.

Grouping the significantly regulated proteins according to their biological functions revealed that proteins were preferentially associated with metabolic process [GO:0008152], carbohydrate metabolic process [GO:0005975], cell redox homeostasis [GO:0045454], translation [GO:0006412], glycogen biosynthetic process [GO:0005978], intracellular protein transport [GO:0006886], protein folding [GO:0006457], glutathione biosynthetic process [GO:0006750], L-phenylalanine catabolic process [GO:0006559] and glycolytic process [GO:0006096] (Fig 7A).

In terms of molecular functions, ATP binding [GO:0005524], zinc ion binding [GO:0008270], RNA binding [GO:0003723], metal ion binding [GO:0046872], GTPase activity [GO:0003924], heme binding [GO:0020037], oxidoreductase activity [GO:0016491], GTP binding [GO:0005525], transferase activity [GO:0016740] and DNA binding [GO:0003677] were the top 10 affected molecular functions (Fig 7B). A comprehensive overview of all gene ontology terms affected by EQ exposure is provided in S8 Table.

### Multi-omics pathway analysis

**Fig 8:**
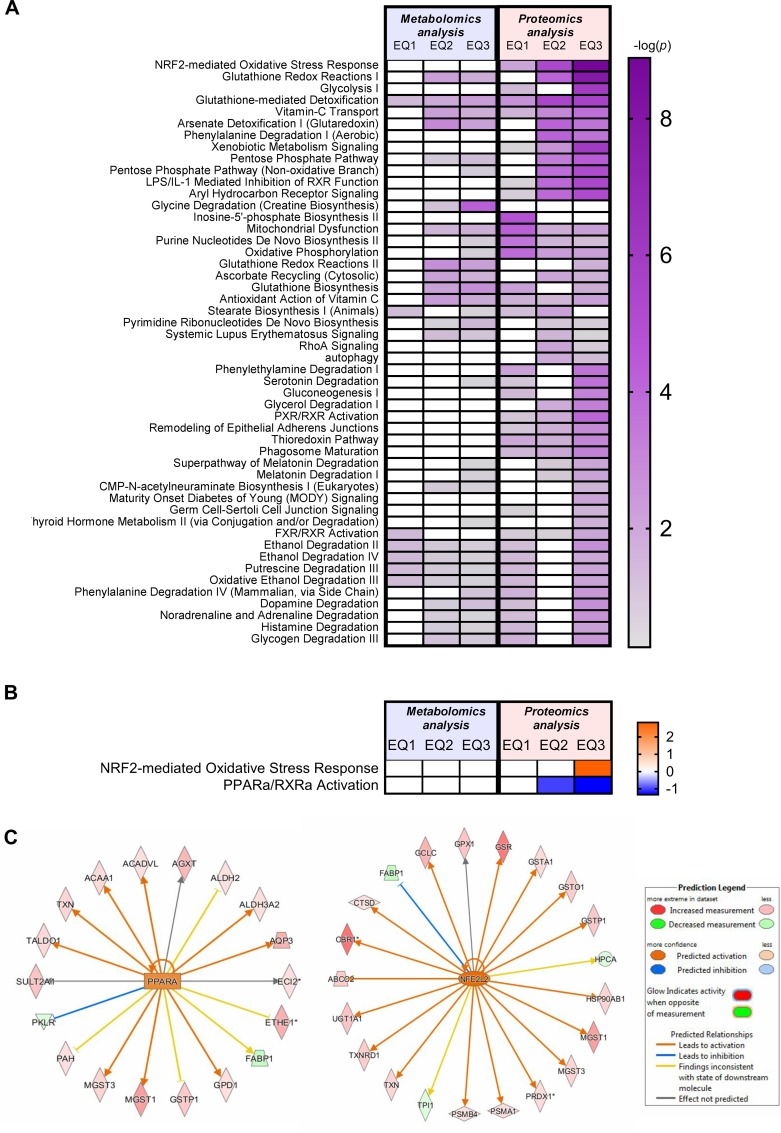
Canonical pathway and upstream regulator analyses of metabolites and proteins in Atlantic salmon (*Salmo salar* L.) exposed to graded levels of ethoxyquin (EQ) through their diet for 90 days. (**A**) Significantly regulated (*p*<0.05, ANOVA) metabolites and proteins in liver subjected to Ingenuity Pathway Analysis (IPA). Statistical significance of overrepresentation of metabolites and proteins in different “canonical pathways” is shown as a heat-map. The top 50 of only selected pathways that obtained a significant (*p*<0.05, Fisher’s exact test) enrichment score (-log10 *p*-value) in at least one of the exposure conditions and were associated with at least two molecules are shown. Scores above the cut-off (1.3) are displayed by a color gradient. Scores below the cut off value are displayed as white boxes. Matching expression patterns of proteins significantly affected in salmon livers after 90 days dietary EQ treatment identified activation (orange) or inhibition (blue) of two canonical pathways **(B)** and highlighted an activation (orange) of NFE2L2 and PPARA as likely affected upstream regulators **(C)** independent of the overlap p-value using the activation z-score. The full data-set including metabolites and proteins in each pathway, the complete list of significant pathways as well as a complete list of predicted upstream regulators are presented in S5A and S10A Tables, and S5D and S10D Tables, respectively.

The Ingenuity Pathway Analysis (IPA) platform was used with default settings to group significantly affected metabolites and proteins into larger functional categories (S5A–S5D Table and S10A–S10D Table, respectively). Integration of data from proteomic and metabolomic data in a comparative analysis revealed distinct and overlapping dose-dependent responses on protein and metabolite level (Fig 8). Comparative analysis of “Canonical pathways” annotations from differentially expressed proteins and altered metabolites confirmed an overlap of a number of enriched pathways, consistently highlighting “NRF2-mediated Oxidative stress Response”, “Glutathione Redox Reactions I”, “Glycolysis”, “Glutathione-mediated detoxification”, “Vitamin C Transport”, “Arsenate detoxification (Glutaredoxin)”, Phenylalanine Degradation (Aerobic)”, “Xenobiotic Metabolism Signaling”, “Pentose Phosphate Pathway”, “Pentose Phosphate Pathway (Non-oxidative Branch)”, LPS/IL-1 Mediated Inhibition of RXR Function” and “Aryl Hydrocarbon Receptor Signaling” as the 10 most affected pathways. Furthermore, the pathway “Purine Nucleotides De Novo Biosynthesis” was consistently associated with EQ-induced dose-dependent responses on metabolite and protein level, while effects on “Pyrimidine Ribonucleotides De Novo Biosynthesis”, “Uridine-5’-phosphate Biosynthesis” and “Salvage Pathways of Pyrimidine Ribonucleotides” were not evident on protein level (Fig 8A).

Causal network analysis of protein expression data, in which the direction of changes was taken into account, highlighted an induction of the “NRF2-mediated Oxidative stress response” at a dose of EQ 3 (z-scores ≥ 1), and a dose-dependent inhibition of “PPARa/RXRa activation” at doses above EQ 1 (z-scores ≤ -1) from changes induced in liver proteins (Fig 8B). These findings were further investigated through upstream analyses in IPA, which predicted a dose-dependent activation of both transcription factors NFE2L2 and PPARA from a significant overlap of EQ-induced changes in proteomic profiles with known responses compiled in the Ingenuity Knowledge Base (Fig 8C).

The full data-set including metabolites and proteins in each canonical pathway, as well as a complete list of predicted upstream regulators are presented in S5A and S10A Tables, and S5D and S10D Tables, respectively.

### Hematology and plasma biochemistry

In order to assess the general health status of the fish as well as the effects of increasing dietary EQ on the immune system, and kidney and spleen as the hematopoietic centers, the number of RBC, Hct, Hb concentration and lysozyme activities were determined, and a differential blood cell count was performed (S12 Table). There were no differences between the groups for any of the above listed parameters.

Plasma biochemical markers of organ function were measured as albumin, ALT, AST, bilirubin, bile acids, total protein, creatinine, Na^+^, K^+^, Ca^2+^, Cl^-^, osmolality and lysozyme activity (Table 4). Ninety days of dietary exposure to graded levels of EQ led to a dose-dependent decrease of plasma creatinine levels where fish exposed to doses above EQ 2 (12.20 ± 2.53 μmol/L) displayed significantly lower levels compared to the Control (22.13 ± 5.62 μmol/L, 45% decrease; *p*<0.01). Plasma bile acid concentrations showed a 2.8-fold increase (*p*<0.05) in animals exposed to the lowest dose (EQ 1; 41 mg/kg feed) when compared to animals with no EQ added to the diets, and further appeared to be inversely related to dietary EQ exposure.

**Table 4:** Plasma biochemical markers of organ function and stress of Atlantic salmon (*Salmo salar* L.) exposed to graded levels of ethoxyquin (EQ) through their diet for 90 days.

	Dietary treatment						
Markers of organ function	EQ 0	EQ 1	EQ 2	EQ 3	EQ 4	EQ 5	P-values
Albumin (umol/l)	344.79 ± 30.02	326.25 ± 24.49	347.29 ± 19.04	362.88 ± 31.21	350.07 ± 35.00	286.94 ± 59.73	< .05
ALT (U/l)	12.81 ± 9.92	14.24 ± 5.74	21.54 ± 13.09	18.84 ± 13.13	13.19 ± 9.25	3.60 ± 2.99	< .05
AST (U/l)	431.78 ± 106.55	459.85 ± 149.48	456.40 ± 85.51	412.63 ± 129.22	411.02 ± 165.32	266.68 ± 110.86	0.54
Bilirubin (umol/l)	1.60 ± 0.32	1.56 ± 0.49	1.41 ± 0.38	1.69 ± 0.33	2.05 ± 0.51	2.06 ± 0.28	< .01
Bile acids (umol/l)	1.80 ± 0.98	5.01 ± 4.14*	4.37 ± 3.81	2.85 ± 2.27	2.51 ± 1.23	1.57 ± 0.31	< .05
Total protein (g/l)	48.04 ± 4.64	45.84 ± 4.61	48.40 ± 3.21	48.17 ± 5.01	46.27 ± 5.23	36.68 ± 7.58*	< .01
Creatinine (umol/l)	22.13 ± 5.62	19.35 ± 5.53	19.29 ± 4.47	12.20 ± 2.53*	9.57 ± 2.38*	8.64 ± 1.64*	< .0001
Na^+^	172.40 ± 3.38	172.20 ± 2.91	171.60 ± 2.17	173.07 ± 3.08	171.40 ± 3.11	172.40 ± 4.22	0.95
K^+^	2.18 ± 0.90	2.25 ± 0.68	2.56 ± 0.98	2.07 ± 0.95	2.12 ± 0.60	1.85 ± 0.80	0.64
Ca^2+^	1.75 ± 0.09	1.78 ± 0.05	1.74 ± 0.08	1.76 ± 0.05	1.76 ± 0.07	1.79 ± 0.11	0.71
Cl^-^	146.93 ± 2.46	147.87 ± 2.88	147.53 ± 1.77	148.13 ± 2.72	146.80 ± 2.27	147.07 ± 3.85	0.75
Osmolality (mOsmol/l)	349.27 ± 12.05	351.07 ± 8.04	350.00 ± 6.53	349.40 ± 9.97	349.33 ± 11.17	344.80 ± 11.55	0.93

Measurements performed on five replicate measurements from the individual tanks (*n* = 3/group). A nested ANOVA was fit using a mixed effects model with tank treated as a random effect, and post-hoc comparison of least-square group means adjusting for multiple comparisons. Results are presented as means ± SD of all measurements.

* *p*<0.05, compared to the control (EQ 0).

Abbreviations: ALT; Alanine aminotransferase. AST; Aspartate aminotransferase.

Other effects observed were lower levels of total protein (*p*<0.05), and the liver enzymes ALT and AST in plasma of fish exposed to the highest dose of EQ (EQ 5; 9666 mg EQ/kg feed) during 90 days compared to the control or the two lowest doses (EQ 1 and EQ 2), respectively. Concomitantly, the concentrations of bilirubin in plasma of fish exposed to doses above EQ 3 (EQ 4: *p* = 0.080 and EQ 5: *p* = 0.094) were elevated. None of these plasma markers were affected by dietary EQ treatment at lower doses. This observation may therefore be a direct result of the general status of malnutrition associated with the reduced feed intake at doses above EQ 3 rather than an effect induced by EQ. There were no differences in plasma electrolyte concentrations or osmolality between any of the groups.

### Liver oxidative stress status

In order to assess the indications of oxidative stress and altered purine/pyrimidine metabolism found in the metabolomics and proteomic screening, targeted analyses of biomarkers of oxidation and oxidative DNA damage were performed. Following 90 days dietary exposure to EQ, changes in hepatic redox homeostasis were noted (Table 5). A significant increase (<25%) in hepatic TBARS concentrations was observed after 90 days exposure to doses above 118.8 mg/kg feed (EQ 3; *p*<0.05), indicating increased rates of lipid peroxidation. Concomitantly, hepatic vitamin E levels were significantly reduced (*p*<0.001). While alpha-tocopherol concentrations significantly decreased (<30%; *p*<0.001) at doses above EQ 3 compared to the control (2330.0 ± 277.8 mg alpha tocopherol/kg), hepatic gamma-tocopherol were already significantly lower (<20%, *p*<0.05) after exposure to the lowest dose of EQ (41 mg EQ/kg feed). Moreover, a substantial increase in the total hepatic glutathione pool was observed. Dietary EQ elevated the levels of both GSH and, to a lower degree, GSSG in a dose-dependent manner (*p*<0.001 and *p*<0.01, respectively). No significant changes were observed in DNA oxidation and oxidative DNA damage as quantified by measurement of 8-oxo-dG formation and number of AP sites.

**Table 5:** Oxidative stress markers and markers for oxidative DNA damage in livers of Atlantic salmon (*Salmo salar* L) exposed to graded levels of ethoxyquin (EQ) through their diet for 90 day.

	Dietary treatment						
	EQ 0	EQ 1	EQ 2	EQ 3	EQ 4	EQ 5	**P-values**
*Oxidative stress*							
GSH (μM)	2445 ± 350	3276 ± 664	3538 ± 613	4805 ± 675*	6431 ± 612*	6141 ± 1765*	< .0001
GSSG (μM)	6 ± 1	7 ± 1	6 ± 1	9 ± 2	12 ± 2*	10 ± 2*	< .01
GSH/GSSG	420 ± 46	491 ± 87	509 ± 97	557 ± 101	574 ± 125	666 ± 178	0.157
TBARS (nmol/g ww)	3.5 ± 0.3	3.5 ± 0.3	3.4 ± 0.1	4.4 ± 0.4*	5.2 ± 0.9*	4.7 ± 0.3*	< .01
*Vitamin E (mg/kg ww)*							
α-tocopherol	2330.0 ± 277.8	2336.7 ± 193.5	2053.3 ± 30.6	1633.3 ± 66.6*	1353.3 ± 121.0*	1193.3 ± 156.3*	< .0001
γ-tocopherol	31.0 ± 7.0	24.7 ± 2.1*	23.3 ± 1.5*	15.9 ± 1.8*	13.1 ± 1.4*	12.3 ± 2.4*	< .0001
*Oxidative DNA damage*							
AP sites/10^5 bp	37.1 ± 4.4	40.0 ± 3.0	30.4 ± 0.4	27.7 ± 7.9	31.2 ± 8.8	32.9 ± 5.6	0.173
8-oxodG (nM)/DNA (μg/μl)	14.2 ± 1.7	14.1 ± 0.7	14.1 ± 0.7	14.1 ± 0.5	13.9 ± 0.8	15.9 ± 1.1	0.214

Measurements performed on tank pooled samples or tank means (*n* = 3/group) were analyzed performing one-way ANOVA and Tukey’s test for multiple comparison of group means. For parameters including replicate measurements from the individual tanks (*n* = 25) a nested ANOVA was fit using a mixed effects model (nlme) with tank treated as a random effect and post-hoc comparison of least-square group means adjusting for multiple comparisons. Results are presented as means ± SD of all measurements.

* *p*<0.05, compared to the control (EQ 0).

Abbreviations: GSH; Glutathione (reduced), GSSG; Glutathione (oxidized), TBARS; Thiobarbituric acid reactive substances, AP; apurinic/apyrimidinic, bp; base pairs, 8-oxodG; 8-hydroxy-2’-deoxyguanosine.

### Gross pathology/histology

In kidney (Table 6), presence of higher number and intensity of pigmented macrophage aggregates (PMA) was observed in fish exposed to the highest dose of EQ (EQ 5, *p*<0.05). Additional Pearl’s Prussian Blue staining of kidney sections verified absence of iron-derived pigments. Thus, the increase in PMA did not appear to be associated to hemolysis (S1A and S1B Fig). Some hyaline drops were observed in tubules, although the number of observed drops was small and thus not considered pathological (S1C and S1D Fig).

**Table 6:** Mean scores for the different histopathological parameters used to assess the kidney, spleen and liver damage in Atlantic salmon (*Salmo salar* L.) exposed to graded levels of ethoxyquin (EQ) through their diet for 90 day.

	Dietary treatment					
Parameter assessed	EQ 0	EQ 1	EQ 2	EQ 3	EQ 4	EQ 5
**Kidney**						
Presence of pigmented macrophages aggregates (PMA)	0.3 ± 0.3	0.2 ± 0.2	0.2 ± 0.2	0.4 ± 0.4	0.4 ± 0.4	1.2 ± 1.2*
**Spleen**						
Congestion and subcapsular hemorrhages	0.7 ± 0.7	0.2 ± 0.3	0.1 ± 0.2	1.0 ± 0.6	1.0 ± 0.5	1.6 ± 0.5*
**Liver**						
Vacuolization in hepatocyte cytoplasm	2.1 ± 0.6	2.1 ± 0.6	2.2 ± 0.8	0.7 ± 0.4*	0.5 ± 0.3*	0.2 ± 0.2*

Scoring was performed in a double-blinded format. Results are presented as mean ± SD of 9 individually scored animals per experimental group.

Group mean scores were compared using Pearson’s X^2^ (level of confidence 95%).

* *p*<0.05. compared to the control (EQ 0).

The main histopathological finding in the splenic tissue was red pulp congestion and subcapsular hemorrhage in fish fed the highest dose of ethoxyquin (EQ 5, *p*<0.05; Table 6 and S2 Fig). However, some of the fish fed the lower EQ levels showed moderate congestion, suggesting that changes observed in splenic tissue were likely not related to EQ treatment.

**Fig 9:**
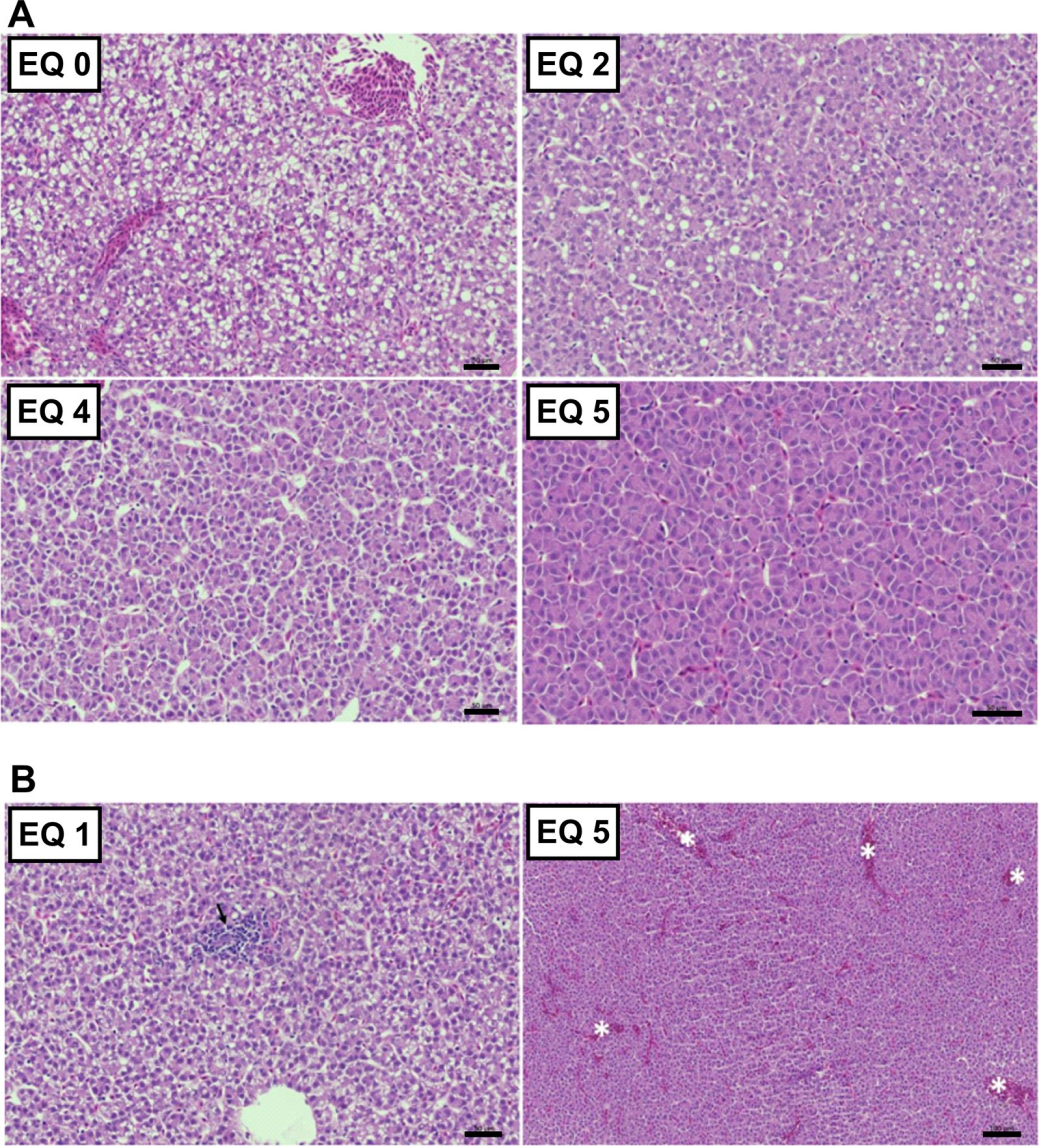
Histopathological findings in of Atlantic salmon (*Salmo salar* L.) exposed to graded levels of ethoxyquin (EQ) through their diet for 90 days. Liver sections (5μm) showed reduced intracytoplasmic vacuolization along with the increase in dietary content of EQ **(A)**. H&E, scale bars: 50 μm. **(B)** Non-treatment related findings included lymphocytic-type infiltrate (left panel, arrow) and hepatic congestion (right panel, asterisk). H&E, scale bars: 50 μm (left panel) and 100 μm (right panel).

Histopathological evaluation of liver tissue revealed a marked reduction in the glycogen and lipid storage along with an increase of dietary EQ concentrations. The decreased hepatocellular vacuolization, a visible reduction in hepatic energy stores, correlated with the reduced feed intake in fish fed high levels of EQ (EQ 4 and EQ 5; Fig 9A). However, a significant decrease in liver cytoplasmic vacuolization was also observed in fish receiving EQ 3 compared to control animals, although this dose did not negatively affect feed consumption. Other changes observed in the liver were mild inflammatory infiltrate (Fig 9B- left panel), mainly lymphocytes, and moderate congestion (Fig 9B- right panel), although these findings seemed casual and not treatment-related. The number of mitosis/apoptosis observed was normal and did not differ between treatment groups.

### Benchmark dose assessment

The BMDL assessment was performed on the estimated daily dose versus biological parameter (Table 7). For fish fed the two highest feed concentrations (3985 and 9666 mg EQ/kg), a significant (*p*<0.05, ANOVA, Tukey’s-*t*) lower feed intake was observed compared the other exposure groups. For biological parameters directly affected by reduced feed intake (growth, feed conversion, organ index) BMDL assessment was performed with the first three exposure groups (EQ 0- EQ 3), which did not differ in feed intake. As reduced growth performance is generally considered an adverse effect, the default BMR of 5% was chosen, and a BMDL_05_ was established at 0.8 mg EQ/kg BW/day for the condition-factor (k). No trend was observed for changes between EQ 0 and EQ 3 for any of the other parameters (AllAIC>AICnull-2).

**Table 7:** Benchmark doses (BMD), lower and upper 90% confidence interval (BMDL and BMDU, respectively) and No Observed (Adverse) Effect Level (NOAEL/NOEL) for relevant responses in Atlantic salmon (*Salmo salar* L.) exposed to graded levels of ethoxyquin (EQ) through their diet for 90 day. Values are given in mg EQ/kg BW/day estimated from feed intake.

	BMD_05_	BMDL_05_	BMDU_05_	BMD_10_	BMDL_10_	BMDU_10_	BMD_20_	BMDL_20_	BMDU_20_	NOAEL (NOEL)
Endpoint										ANOVA
Growth parameters (EQ 0 –EQ 3)										
Condition factor (k)	11.4	0.8	121.0							11
Hematological parameters (EQ 0 –EQ 5)										
Hct	28.2	13.1	40.0							
HGB	26.8	14.9	36.0							
MCV	7.4	1.1	63.8							
MCH	28.8	2.6	80.3							
Biomarkers of liver function (EQ 0- EQ 5)										
Bile acids	none	none	none				none	none	none	0.4
ALT							26.4	14.5	32.5	n.s. (30)
AST				25.8	10.9	29.3				n.s. (30)
Bilirubin							17.8	5.5	30.5	n.s. (11)
Albumin							45.1	41.5	49.7	30 (p = 0.058)
Total protein				31.6	28.7	33.6				30
Creatinine							2.0	0.5	9.2	1.2
Liver oxidative stress markers (EQ 0- EQ 5)										
TBARS							8.0	2.3	13.1	1.2
Liver alpha-tocopherol							4.6	1.7	9.2	1.2
Markers of oxidative DNA damage (EQ 0- EQ 5)										
8-oxo-dG	none	none	none							n.s. (40)
AP sites	none	none	none							n.s. (40)
Histopathology (EQ 0- EQ 5)										
	Liver: Depletion of vacuoles			6.0	3.2	17.9				11
	Kidney: Severe presence of pigmented macrophage aggregates			38.4	36.7	39.3				30
	Spleen: Severe congestion of spleen tissue			12.1	2.7	35.8				30

Taking the width of the 90% confidence intervals (BMDU/BMDL) into consideration, benchmark responses were set at the default of 5%, or adjusted to 10 or 20%. For derivation of the critical BMDL, the width of the confidence interval was taken into account as an indicator of precision. The No-observed-(adverse)-effect-levels (NOAEL/NOEL) was determined from statistical comparisons of responses in each predetermined dose group with the control group. Raw data and the Akaike Information Criteria for all models assessed, as well as models for the critical endpoints included into the evaluation for the critical BMDL are available in S16 Table.

Abbreviations: Hct, Hematocrit; HGB, Hemoglobin; MCV, Mean corpuscular volume; MCH, Mean corpuscular hemoglobin; ALT, Alanine amino-transferase; AST, Aspartate aminotransferase; TBARS, Thiobarbituric acid reactive substances; GSH, Reduced glutathione; GSSG, Oxidized glutathione; 8-oxo-dG, 8-hydroxy-2’-deoxyguanosine; AP, apurinic/apyrimidinic.

For blood and plasma parameters, that are less likely to be directly affected by reduced feed intake (no starvation occurred) a BMDL was assessed including all exposure groups. Although ANOVA did not detect significant effects for any of the hematological parameters, models could be fit to Hct, HGB, MCV and MCH, with BMDL_05_ ranging between 1.1 (MCV) and 14.9 (HGB) mg EQ/kg BW/day. Because the model fit for some of the plasma markers indicated a poor precision of the estimates (as indicated by broad confidence intervals), the BMR was adjusted to 10% above the modelled background response for AST and plasma total protein, and a 20% was used for ALT, bilirubin, plasma albumin, and plasma creatinine. Of plasma markers of physiological/metabolic effects, creatinine had the lowest BMDL_20_ (0.5 mg EQ/kg BW/day). However, as creatinine levels directly correlate with energy turn-over, the observed effect could be the results of reduced feed intake rather than effects of EQ. The lowest BMDL_20_ for plasma markers of adverse effects was therefore set for bilirubin with a BMDL_20_ of 5.5 mg EQ/kg BW/day. Typical plasma markers of adverse effects on liver such as liver enzymes ALT and AST had a BMDL_20_ of 14.5 mg EQ/kg BW/day and BMDL_10_ of 10.9 mg EQ/kg BW/day, respectively (Table 7). For liver TBARS, a measurement of formation of potentially toxic lipid peroxidation products, a BMDL_20_ of 8.0 mg EQ/kg BW/day was established (Table 7).

Furthermore, changes in liver GSH and GSSG levels as well as liver vitamin E levels were assessed. Changes in the liver oxidative defense systems are considered homeostatic responses and were as such regarded as markers of exposure rather than markers of adverse effect. As expected, as effect of exposure the BMDL_20_’s for these parameters were very low. With 0.0008 mg/ kg BW/day, liver GSSG levels had the lowest BMDL_20_ of the adaptive oxidative stress responses. However, a very large BMDU/BMDL (> 20 000) indicated an unreliable estimate (S16 Table). With a BMDU/BMDL of 5.4, alpha tocopherol in the liver showed the most reliable assessment for the liver adaptive responses and a BMDL_20_ of 1.7 mg EQ/kg BW/day was established. No trend was observed for the markers of oxidative damage (8-oxo-dG and AP sites; AllAIC>AICnull-2).

For histology data a BMDL was derived from an average model constructed by averaging all weighted-model results of the default set of models run. With 2.7 mg EQ/kg BW/day the lowest BMDL_10_ was established for “Severe congestion of spleen tissue” (Table 7). However, the BMDL_10_ of 3.2 mg EQ/kg BW/day “Severe depletion of intracytoplasmic vacuoles” in liver was found the most reliable assessment (BMDU/BMDL: 5.5). A complete overview of the raw data used for the analysis, all modelled parameters and the models included into the evaluation of the critical BMDL, is provided in S14–S16 Tables.

## Discussion

Ethoxyquin is widely used as an additive in ingredients and formulated feeds for farmed animals including Atlantic salmon. The toxic potential of EQ in salmon has yet to be addressed. The present study investigated effects of dietary EQ in Atlantic salmon through a 90-day subchronic dose-response study, using a dose range from 0 to 10 000 mg EQ/ kg complete feed.

Dietary exposure to EQ at concentrations above 1173 mg EQ/kg feed, namely EQ 4 (3985 mg EQ/kg) and EQ 5 (9666mg EQ/kg) significantly affected feed intake and growth performance in Atlantic salmon (Table 3). Similar to our findings, Bohne et al. [5] found no effects of dietary EQ on growth or feed intake in Atlantic salmon up to a concentration of 1800 mg/kg feed, whereas diets containing a concentration of 15 000 mg/kg feed were rejected by the fish. Since all animals included in the present study came from the same stock and were reared under identical experimental conditions, the differences in feed intake and growth performance can most likely be attributed to dietary EQ levels as the only variable factor. Previous studies have reported inconsistent findings of the effects of dietary EQ exposure on growth response. Improved growth, as observed by several studies on EQ in different animal species [29,30,31,32], may likely be due to preserved nutritional quality of the diet under storage, especially prevention of lipid oxidation by EQ [29], which may affect palatability of aquafeed. On the other hand, although preventing lipid oxidation in stored diets, inclusion of 200 mg EQ/kg feed led to reduced growth in shrimp, which was associated with reduced feed intake and altered feeding behavior [33]. Similarly, reduced growth rates in juvenile large yellow croaker (*Pseudosciaena crocea)* after exposure to 1350 mg EQ/kg feed were associated with a trend in decreased feed intake with increasing dietary EQ, although no significant effect was observed [11].

As EQ is described to have a mercaptan like scent [10], inclusion of high levels of EQ may have affected diet palatability due to its sensory properties. Indeed, fish receiving diets containing 3985 (EQ 4) and 9666 (EQ 5) mg EQ/kg consumed only 60% and 13% compared to the feed intake of the control group (EQ 0) already during the first week indicating that poor palatability prevented the fish from consuming the diet. Alternatively, altered feeding behavior at higher doses could also be secondary to acute effects of EQ, e.g. due to organ damage caused by EQ exposure. However, there were no mortalities linked to any dietary treatment during the study.

Excluding EQ 4 and EQ 5 exposed fish and focusing on dietary groups with similar feed intake, integration of data from proteomic and metabolomic screenings revealed distinct and consistent EQ-induced responses in livers of salmon exposed to 119 (EQ 2) and 1173 (EQ 3) mg EQ/kg feed compared to the control group (EQ 0); these were mainly related to changes in energy metabolism, redox homeostasis and purine/pyrimidine metabolism. For other effects, see S5A–S5C and S10A–S10C Tables. Observed alterations in the fatty acid metabolism, such as e.g. reduced levels of diacylglycerols and carnitine, and elevated acetyl CoA (Fig 2), suggested increased fatty acid β-oxidation in fish exposed to EQ at doses higher than 1.22 mg/kg BW/day (EQ 2). The indications from altered metabolite levels were further supported by the bioinformatic pathway analysis of EQ-induced changes on the liver protein levels using IPA, which predicted an activation of peroxisome proliferator-activated receptor alpha (PPARα), the master regulator of hepatic fatty acid β-oxidation, from a significant overlap in the response compared to known targets (Fig 8C), as well as (PPARα)/retinoid X receptor alpha (RXRα) activation as an affected canonical pathway (Fig 8B). Furthermore, changes in individual metabolite levels displayed an elevation of glucose 6-phosphate and reduced phosphoenolpyruvate, along with significant increases in other glycolytic intermediates (Fig 3A). Consistently, functional annotations of altered protein levels in liver were preferentially related to metabolic processes as well as carbohydrate metabolic processes (Fig 7A). The observed changes may reflect a mobilization of hepatic glycogen stores but may also be due to a redirection of glucose to the pentose phosphate pathway, as revealed by the performed pathway analysis (Fig 8A).

The pentose phosphate pathway utilizes glucose 6-phosphate to generate NADPH for biosynthetic reactions and Phase I metabolism of xenobiotics through the cytochrome P450 system. Indeed, induction of CYP1A1 and CYP3A mRNA was affected by dietary EQ indicating Phase I metabolism of EQ as shown by Bohne et al. [14]. The decrease in pentose sugars and some of the pentose phosphate pathway intermediates may be indicative of a decrease in glucose supply or increased pathway activity to support the demand for NADPH that can be used to support detoxification reactions, which were consistently associated with the observed alterations in metabolite and protein levels in livers of EQ exposed salmon in the present study (Fig 8A). Moreover, consistent with the effect of EQ treatment observed on plasma creatinine levels (Table 4), hepatic creatinine levels decreased in a dose-dependent manner. Lowered plasma creatinine levels are commonly used as biomarker for renal dysfunction, but may also reflect declining hepatic functional capacity and alterations in hepatic energy metabolism. Taken together, the results from metabolomic and proteomic screening indicated that an increased energy expenditure in response to EQ exposure, perhaps due to the costs of detoxification or tissue repair [34].

Organ damage resulting from EQ exposure has previously been described for many different animals. High concentrations of EQ metabolites have been found in the liver of Atlantic salmon fed graded levels of EQ [35]. Indeed, EQ readily accumulates in the liver, which was shown to be a main target site of EQ-induced adverse effects in other species, as described in a Scientific opinion on safety and efficacy of EQ for all animal species published by EFSA in 2015 [8]. In fish, reduced hepatosomatic indices were reported for large yellow croaker receiving 450 and 1350 mg EQ/kg feed for 10 weeks [11], while Saxena et al. [12] found that dietary exposure to 200 and 400 mg EQ/kg feed for 16 days increased the hepatosomatic index in turbot. Similar to previous findings in Atlantic salmon by Bohne et al. [14], in the present study EQ did not affect liver, spleen or heart weights relative to body size after 90 days exposure with the exception of fish exposed to EQ 5, which had a low feed intake throughout the study.

As seen from the histological evaluation of liver, kidney, and spleen in the present study, liver seemed to be the main organ for EQ toxicity in Atlantic salmon. Despite the absence of clear EQ-induced changes in relative liver weights, histological evaluation of liver sections in the present study showed a dose-dependent decrease in intracytoplasmic vacuolization at exposure to feed concentrations above 119 mg EQ/kg (EQ 2), which indicated a loss of cytoplasmic glycogen and/or lipid. While livertoxicity is more commonly associated with increased hepatocellular vacuolization [36], the observed histopathological changes with a depletion of hepatocellular vacuoles were consistent with the induction of lipid and carbohydrate catabolic pathways in groups exposed to 119 (EQ 2) and 1173 (EQ 3) mg EQ/kg feed compared to the control group (EQ 0) as indicated from the results of the metabolomic/proteomic screening. Elevated energy expenditure in response to dietary EQ exposure was further corroborated by the observed dose-dependent decrease in lipid content in whole fish at doses above 119 mg EQ/kg (EQ 2). Other changes observed in the liver were moderate congestion and mild inflammatory infiltrate (mainly lymphocytes; Fig 9B), although these findings seemed casual and not treatment-related. The number of mitosis/apoptosis observed was within the normal range and did not appear to be affected by the treatment, which does not seem to indicate increased cytotoxicity by EQ as previously described *in vitro* [37].

The metabolomic and proteomic screening indicated changes associated with redox-homeostasis mediated through increased utilization of glutathione (GSH). GSH is an important endogenous antioxidant that participates both directly and indirectly in the scavenging of reactive oxygen species (ROS). GSH may react directly with ROS with subsequent formation of glutathionyl radicals, but is also an electron donor for antioxidant enzymes such as glutathione peroxidase where GSH is oxidized to GSSG. To maintain the levels of reduced GSH for continued antioxidant defense, a redox cycle is formed where GSSG is reduced back to GSH by glutathione reductase using NADPH [38]. In line with previous evidence from a study in mice [39], the canonical pathway analysis (Fig 8) of salmon liver metabolome and proteome suggested that EQ activates the NRF2 (Nuclear factor erythroid 2-related factor 2) signaling pathway, resulting, primarily, in an increase in the transcription of detoxifying enzymes such as certain glutathione-S transferase (GSTs) isoenzymes or NAD(P)H: quinone oxidoreductase 1 (NQO1).

Quantitative analyses of GSH and GSSG (Table 5) confirmed increased concentrations of GSH in livers of fish exposed to EQ at concentrations higher than 119 mg/kg feed (EQ 2), while increased concentrations of GSSG were observed in fish exposed to concentrations higher than 1173 mg EQ/kg feed (EQ 3). GSH is synthesized *de novo* via the SAM cycle and the trans-sulfuration pathway from the amino acids cysteine, glutamate and glycine. The first step is catalyzed by glutamate-cysteine ligase (GCL). Oxidative stress increases the GCL activity to produce more GSH [38,40,41]. Although a depletion of cellular GSH storage is more commonly used as a marker for oxidative stress, the observed shift in the cellular redox equilibrium may reflect compensatory induction of GSH biosynthesis and thus indicate a situation of oxidative stress. Indeed, a dose-dependent depletion of vitamin E was seen for EQ doses above 119 mg EQ/kg (EQ 2) for α-tocopherol and above 0.5 mg EQ/kg (EQ 0) for γ-tocopherol. A consequence of reduced levels of vitamin E is a reduced capacity to protect against oxidative damage to DNA, proteins and lipids. A common method of detecting oxidative damage to lipids is by determining products of lipid peroxidation such as malondialdehyde (MDA) using the TBARS assay [42]. Elevated levels of TBARS were found for EQ doses above 119 mg EQ/kg (EQ 2) indicating increased levels of MDA, though the TBARS method has been criticized for having low specificity for MDA detection [42]. Nevertheless, the findings were consistent with other markers of oxidative stress.

MDA has the capacity to impair several physiological mechanisms of the human body through its ability to react with molecules such as DNA and proteins. MDA can react with DNA and form nucleic acid adducts and are associated with a variety of pathological events [43]. In the metabolomic screening, alterations in the purine and pyrimidine metabolism were found for all dietary inclusion levels of EQ for adenine (Fig 5A). These alterations in pyrimidine and purine metabolism could imply an increased requirement for nucleotides for DNA or RNA repair due to oxidative stress. The formation of 8-hydroxy-2’-deoxyguanosine (8-oxo-dG) is considered a predominant DNA alteration in oxidative DNA damage [44]. Moreover, DNA damage can manifest in the formation of apurinic or apyrimidinic (AP or abasic) sites. EQ has been shown to induce chromosome aberrations including breaks, dicentrics, atypical translocated chromosomes and chromatid exchanges *in vitro* [37,45,46,47]. However, in the present study no significant changes were observed in DNA oxidation in either of the two included biomarkers of DNA damage (Table 5). On the other hand, adenine is also a precursor of several intermediates involved in energy metabolism; ATP, FAD, NADH and NADPH. However, the derived structures adenosine, AMP and ADP were not elevated in a dose-dependent manner and thus appeared not-treatment related. Conversely, the mono- and di-phosporylated forms of guanine nucleotide (GTP), which also serves as an energy source for protein synthesis and gluconeogenesis, were decreased at dose levels above EQ 1, but not for EQ 5. Orotate, a precursor in the biosynthesis of pyrimidines, was elevated at doses above EQ 0, but no clear dose-related pattern could be seen for cytidine and uracil containing pyrimidines. Taken together, these changes could be related to the direct effects of EQ on purine and pyrimidine synthesis but might also be related to the altered activity of pentose phosphate pathway (Fig 8A). Given the observed changes in purine/pyrimidine metabolism, independent studies should address if these changes are due to genotoxic effects of EQ, increased cell proliferation, changes in repair mechanism, or are secondary to alteration in pentose phosphate pathway activity.

### Benchmark dose assessment and derivation of a safe upper intake level

The benchmark dose (BMD) approach was used to characterize toxicity risk and derive a reference point for the upper level of intake of EQ for salmon. Using the experimental data to model a dose-response relationship for an adverse outcome, low but detectable increases can be used to determine the dose threshold for a toxic effect. When deriving a reference point, the EFSA recommends to replace the traditionally used no-observed-adverse-effect level (NOAEL) with the 90% lower confidence limit of the BMD model (BMDL) [15].

In addition to the histopathological findings, metabolomic and proteomic screenings and a range of biomarkers were measured to evaluate the toxic mode of action for EQ. In 2017, the EFSA published a new guidance document, in which the difference between adverse effect, biomarkers of exposure or effect, and mode of action (MOA) were defined [48]. The use of biomarkers of exposure in an adverse effect assessement should be assessed cautiously since changes in biomarkers of effect do not necessarily reflect an adverse effect, but rather a result of homeostatic regulation or adaptation [49]. This is particularly true for–omics data; although useful for gaining new insights in mechanistic toxicology and generating hypotheses about disease pathways, their semi-quantitative nature calls for a conservative approach when using them in assessing severity of effects [49]. For this reason, BMD modelling was not performed for the–omics data. The use of biomarkers of effects that are related to EQ specific modes of toxic actions and occur prior to the development of overall adverse effect, are however relavant to use in assessing adverse effects in subchronic (30% of life time; 3 months) exposure studies.

The most significant responses observed in the present study were a perturbation of energy metabolism, induction of oxidative stress and altered purine/pyrimidine metabolism. All quantitative biomarkers, possibly representing an impairment of functional capacity (i.e. the hematological parameters, biomarkers of liver function and the biomarkers of oxidative stress), were subjected to BMD modelling. However, representing a manifestation of physiological effects, effects on the growth response and the morphological changes observed in the histopathological evaluation of spleen, kidney and liver in the present study were regarded the most biologically relevant effects [48], and thus considered as critical endpoints for this study (Table 7). Although the lowest BMDL was estimated for a decreased condition factor (k) (BMDL_05_: 0.8 mg EQ/kg BW/day), in addition to its high biological relevance, the „severe depletion of intracytoplasmic vacuoles”observed in the liver had the lowest BMDU/BMDL, and was thus the most reliable BMDL estimate. Hence, the BMDL_10_ of 3.2 mg EQ/kg BW/day was considered the critical BMDL and is proposed as reference point for a safe upper exposure level of EQ in Atlantic salmon.

## Conclusion

The results from the present study indicated that subchronic dietary exposure to concentrations above 119 mg EQ/kg feed perturb hepatic lipid and carbohydrate metabolism, leading to a depletion of hepatic energy stores, which was associated with alterations in purine/pyrimidine metabolism in Atlantic salmon. EQ-induced effects were associated with presence of oxidative stress in the liver and activation of the NRF2-mediated oxidative stress response.

Although some changes in adaptive redox biomarkers were observed at lower doses, no signs of adverse effects were observed in Atlantic salmon exposed to feed concentrations below 119 mg EQ/kg feed. A critical BMDL_10_ of 3.2 mg EQ/kg BW/day was established for the depletion of intracytoplasmic vacuoles in the liver as the critical endpoint in this study. The BMDL_10_ corresponds to a feed concentration of around 240 mg EQ/kg. However, maximum surveyed EQ levels in fish feed show that this concentration is within the range of detected levels, thus warranting the control of the level EQ supplementation.

## Supporting information

S1 Fig Histopathological findings in kidney sections from Atlantic salmon (*Salmo salar* L.) exposed to ethoxyquin (EQ) through their diet for 90 days. Animals exposed to 0.47 mg EQ/kg (EQ 0; A) and 9666 mg EQ/kg (EQ 5; B) show presence of pigmented macrophages aggregates (PMAs) to a different degree. H&E staining, scale bars: 200 μm. Staining with either H&E (C) or periodic acid-Schiff (D) show the presence of a hyaline drop (arrow) in a fish fed EQ0. Scale bars: 50 μm.
(DOCX)

S2 Fig Histopathological findings in spleen sections from Atlantic salmon (*Salmo salar* L.) exposed to ethoxyquin (EQ) at 9666 mg EQ/kg through the diet for 90 days. The spleen shows marked subcapsular hemorrhage (arrow) and congestion of the red pulp (asterisk). H&E. Scale bar: 200 μm.
(DOCX)

S1 Table Mean concentration of ethoxyquin (EQ; μg/kg ww) and ethoxyquin dimer (EQDM; μg/kg ww) in whole body homogenates and muscle samples of Atlantic salmon (*Salmo salar* L.) exposed to graded levels of EQ through their diet for 90 days. (DOCX)

S2 Table Original scale metabolite profile of liver samples from Atlantic salmon after 90 days dietary exposure to increasing levels of ethoxyquin (EQ) (Metabolon). (XLSX)

S3 Table Liver metabolites significantly altered by dietary treatment with increasing levels of EQ (*p*<0.05; 1wANOVA; Qlucore). (XLSX)

S4 Table Liver metabolites significantly altered compared to EQ0 (*p*<0.05; 1wANOVA and *p*<0.05 planned contrasts vs EQ0; Qlucore). (XLSX)

S5 Table Ingenuity pathway analyses (IPA) of ethoxyquin (EQ)-induced changes on liver metabolites in Atlantic salmon. A) 'Canonical Pathways', B) 'Diseases and Bio Functions', and C) 'Tox Functions' enriched with regulated (*p*<0.05, Qlucore) metabolites in liver samples from Atlantic salmon after 90 days dietary exposure to increasing levels of EQ. D) 'Upstream Regulators' predicted from regulated (*p*<0.05, Qlucore) metabolites in liver samples of Atlantic salmon after 90 days dietary exposure to increasing levels of EQ.
(XLSX)

S6 Table Original scale protein profile of liver samples from Atlantic salmon after 90 days dietary exposure to increasing levels of ethoxyquin (EQ) (PROBE). MaxQuant parameters and Qlucore output.
(XLSX)

S7 Table Uniprot gene ontology terms and keywords of all proteins detected in dataset. (XLSX)

S8 Table Protein groups according to their respective GO terms. (XLSX)

S9 Table Liver proteins significantly altered compared to EQ0 (*p*<0.05; 1wANOVA and *p*<0.05 post-hoc; Qlucore; including entries of mammalian orthologs). (XLSX)

S10 Table Ingenuity pathway analyses (IPA) of ethoxyquin (EQ)-induced changes on liver proteins in Atlantic salmon. A) 'Canonical Pathways', B) 'Diseases and Bio Functions' and C) 'Tox Functions' enriched with regulated (*p*<0.05, Qlucore) proteins in liver samples from Atlantic salmon after 90 days dietary exposure to increasing levels of EQ. D) 'Upstream Regulators' predicted from regulated (*p*<0.05, Qlucore) proteins in liver samples from Atlantic salmon after 90 days dietary exposure to increasing levels of EQ.
(XLSX)

S11 Table Raw data of growth parameters, body composition, organ weights, organ-somatic indices, blood and plasma measurements and measures of oxidative stress and oxidative DNA damage in livers of Atlantic salmon after 45 (T1) and 90 (T2) days exposure to increasing levels of ethoxyquin. (XLSX)

S12 Table Hematological parameters of Atlantic salmon (*Salmo salar* L.) exposed to graded levels of EQ through their diet for 90 days. (DOCX)

S13 Table Raw data of the histological evaluation of livers, spleens and kidneys of Atlantic salmon after 90 days dietary exposure to increasing levels of ethoxyquin (EQ). (XLSX)

S14 Table Raw data for BMDL analysis of critical histological outcomes in liver, spleen and kidneys of Atlantic salmon after 90 days dietary exposure to increasing levels of ethoxyquin (EQ). Scores in the critical categories were translated into quantal data for analysis. (XLSX)

S15 Table Akaike information criteria of benchmark dose model fittings and lower and upper benchmark dose 90% confidence intervals (BMDLs and BMDUs, respectively) of all measures assessed through benchmark dose modelling. (XLSX)

S16 Table Summary of lower and upper bound benchmark dose 90% confidence intervals (BMDLs and BMDUs, respectively), and models of critical endpoints used in the evaluation for a critical BMDL. (XLSX)
