# Supplementary figures and images for: Tolerance and dose-response assessment of subchronic dietary ethoxyquin exposure in Atlantic salmon (Salmo salar L.)
Source: PLoS One. 2019 Jan 25;14(1):e0211128. doi: 10.1371/journal.pone.0211128 (PMC6347454; doi:10.1371/journal.pone.0211128)

**Fig S1**


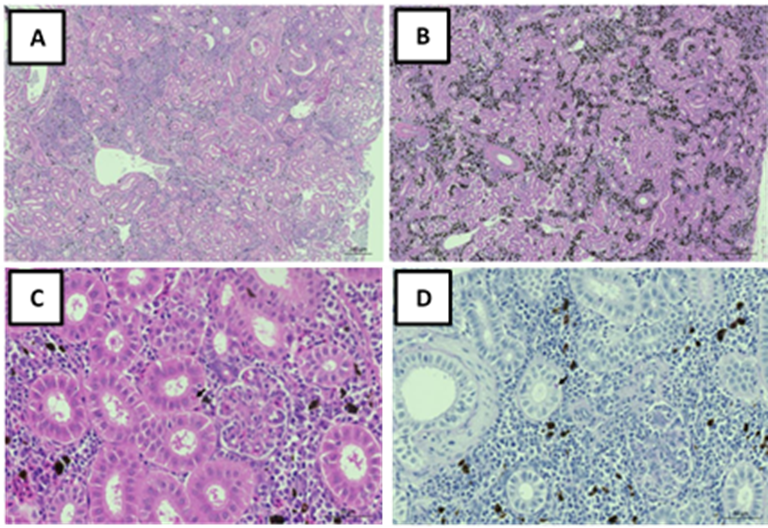

Supplement: S1 Fig — Animals exposed to 0.47 mg EQ/kg (EQ 0; A) and 9666 mg EQ/kg (EQ 5; B) show presence of pigmented macrophages aggregates (PMAs) to a different degree. H&E staining, scale bars: 200 μm. Staining with either H&E (C) or periodic acid-Schiff (D) show the presence of a hyaline drop (arrow) in a fish fed EQ0. Scale bars: 50 μm. (DOCX) [file pone.0211128.s001.docx]

**Fig S2**


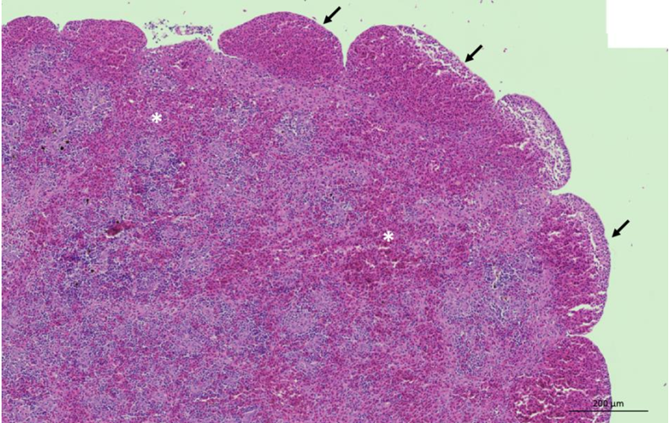

Supplement: S2 Fig — The spleen shows marked subcapsular hemorrhage (arrow) and congestion of the red pulp (asterisk). H&E. Scale bar: 200 μm. (DOCX) [file pone.0211128.s002.docx]
