# Supplementary material for: Tolerance and dose-response assessment of subchronic dietary ethoxyquin exposure in Atlantic salmon (Salmo salar L.)
Source: PLoS One. 2019 Jan 25;14(1):e0211128. doi: 10.1371/journal.pone.0211128 (PMC6347454; doi:10.1371/journal.pone.0211128)
Supplement: S1 Table — (DOCX) [file pone.0211128.s003.docx]

**Table S1.** Mean concentration of ethoxyquin (EQ; µg/kg ww) and ethoxyquin dimer (EQDM; µg/kg ww) in whole body homogenates and muscle samples of Atlantic salmon (*Salmo salar* L.) exposed to graded levels of EQ through their diet for 90 days.

|  | Whole body homogenates | | |  | Muscle | | |
| --- | --- | --- | --- | --- | --- | --- | --- |
|  | EQ |  | EQDM |  | EQ |  | EQDM |
| EQ 0 | 6 ± 3 |  | 386 ± 124 |  | 6 ± 2 |  | 136 ± 70 |
| EQ 1 | 57 ± 10 |  | 351 ± 37 |  | 40 ± 4 |  | 182 ± 32 |
| EQ 2 | 104 ± 51 |  | 328 ± 55 |  | 96 ± 18 |  | 160 ± 59 |
| EQ 3 | 583 ± 173 |  | 648 ± 192 |  | 523 ± 378 |  | 336 ± 126 |
| EQ 4 | 2028 ± 99 |  | 1037 ± 403 |  | 469 ± 365 |  | 709 ± 228 |
| EQ 5 | 3810 ± 483 |  | 1848 ± 962 |  | 539 ± 422 |  | 868 ± 734 |

Measurements performed on tank pooled samples or tank means (n=3/group). Data are presented as mean ± SD.
