# Supplementary material for: Tolerance and dose-response assessment of subchronic dietary ethoxyquin exposure in Atlantic salmon (Salmo salar L.)
Source: PLoS One. 2019 Jan 25;14(1):e0211128. doi: 10.1371/journal.pone.0211128 (PMC6347454; doi:10.1371/journal.pone.0211128)
Supplement: S12 Table — (DOCX) [file pone.0211128.s014.docx]

**Table S12. Hematological parameters of Atlantic salmon (Salmo salar L.) exposed to graded levels of EQ through their diet for 90 days.**

|  | Dietary treatment | | | | | | |
| --- | --- | --- | --- | --- | --- | --- | --- |
|  | EQ 0 | EQ 1 | EQ 2 | EQ 3 | EQ 4 | EQ 5 | **P-values** |
| Erythrocytes (*10^12^/l) | 1.56 ± 0.09 | 1.56 ± 0.22 | 1.59 ± 0.12 | 1.60 ± 0.10 | 1.53 ± 0.14 | 1.54 ± 0.15 | 0.911 |
| Hct (%) | 51.07 ± 2.60 | 48.90 ± 3.30 | 49.57 ± 3.80 | 49.90 ± 3.08 | 47.37 ± 3.49 | 45.40 ± 6.44 | 0.188 |
| Hb (g/dl) | 11.28 ± 0.79 | 11.02 ± 0.71 | 11.17 ± 0.55 | 11.07 ± 0.85 | 10.43 ± 0.80 | 10.23 ± 1.25 | 0.164 |
| MCV | 326.95 ± 10.10 | 318.23 ± 36.46 | 313.28 ± 19.18 | 312.67 ± 11.40 | 311.47 ± 26.61 | 294.26 ± 20.52 | 0.135 |
| MCH | 72.15 ± 2.52 | 71.89 ± 9.74 | 70.71 ± 3.91 | 69.29 ± 3.04 | 68.57 ± 6.12 | 66.48 ± 4.32 | 0.517 |
| MCHC | 22.08 ± 0.87 | 22.56 ± 0.86 | 22.60 ± 1.06 | 22.17 ± 0.86 | 22.02 ± 0.60 | 22.63 ± 1.24 | 0.857 |
| Lysozyme (mg/l) | 0.23 ± 0.09 | 0.19 ± 0.09 | 0.24 ± 0.07 | 0.22 ± 0.09 | 0.18 ± 0.08 | 0.22 ± 0.10 | 0.91 |
| *Differential white blood cell count* | |  |  |  |  |  |  |
| Lymphocytes (%) | 83.60 ± 12.43 | 86.67 ± 7.07 | 83.56 ± 6.00 | 83.00 ± 8.47 | 80.22 ± 6.32 | 80.56 ± 9.84 | 0.794 |
| Neutrophils (%) | 13.90 ± 10.73 | 12.11 ± 7.03 | 14.22 ± 5.59 | 14.44 ± 7.57 | 16.00 ± 5.75 | 16.67 ± 8.96 | 0.891 |
| Monocytes (%) | 2.50 ± 2.95 | 1.22 ± 1.64 | 2.22 ± 2.11 | 2.56 ± 2.60 | 3.78 ± 2.86 | 2.78 ± 2.44 | 0.673 |

Measurements performed on replicate measurements from the individual tanks. A nested ANOVA was fit using a mixed effects model (nlme) with tank treated as a random effect. and post-hoc comparison of least-square group means adjusting for multiple comparisons. Results are presented as means ± SD of all measurements.

Abbreviations: Hct; Hematocrit. Hb; Hemoglobin. MCV; Mean corpuscular volume. MCH; Mean corpuscular hemoglobin. MCHC; Mean corpuscular hemoglobin concentration.
